# Revision of the Genus *Ranacris* You & Lin, 1983 (Orthoptera: Acrididae: Catantopinae), with Proposal of a New Synonym

**DOI:** 10.3390/insects17030298

**Published:** 2026-03-09

**Authors:** Zhongke Lv, Jun Cai, Benyong Mao, Xun Wang, Hong Song, JoVonn G. Hill, Xiongyan Yin, Hanqiang Wang, Jianhua Huang

**Affiliations:** 1 Key Laboratory of Forest Bio-Resources and Integrated Pest Management for Higher Education in Hunan Province, Central South University of Forestry and Technology, Changsha 410004, China; lvzhongke666@gmail.com (Z.L.); caidada910@gmail.com (J.C.); 2 College of Agriculture and Biological Science, Dali University, Dali 671003, China; maoby65@sohu.com (B.M.); fslaaaaa@163.com (H.S.); 3 Mississippi Entomological Museum, Department of Molecular Biology, Biochemistry, Entomology and Plant Pathology, Mississippi State University, Starkville, MS 39759, USA; jgh4@msstate.edu; 4 College of Life Sciences, Shaanxi Normal University, Xi’an 710119, China; 5 Shanghai Entomological Museum, Chinese Academy of Sciences, Shanghai 200032, China; whq@cemps.ac.cn

**Keywords:** *Ranacris*, Acrididae, redescription, new synonym

## Abstract

*Ranacris* You & Lin, 1983, is a small genus in the subfamily Habrocneminae with an undefined phylogenetic position, exhibiting in previous molecular studies a distant relationship with the genera in the same subfamily but an extremely close relationship with the genus *Menglacris* Jiang & Zheng, 1994, in another subfamily, Catantopinae. In this study, a taxonomic revision of *Ranacris* was conducted based on the examination of types and non-type material and the comparison of qualitative and quantitative characters. A morphometric analysis was carried out to test the statistical significance of the quantitative distinguishing characters among *Ranacris*. A new junior synonym is proposed: *R. yunnanensis* Mao, Ren & Ou, 2011 = *R. jinpingensis* Zheng, Lin, Deng & Shi, 2015, **syn. nov**.

## Simple Summary

The genus *Ranacris* You & Lin, 1983, is revised based on the examination of types and non-type material and the comparison of qualitative and quantitative characters. A new junior synonym is proposed: *R. yunnanensis* Mao, Ren & Ou, 2011 = *R. jinpingensis* Zheng, Lin, Deng & Shi, 2015, **syn. nov**.

## 1. Introduction

*Ranacris* You & Lin, 1983, was established with *R. albicornis* You & Lin, 1983, as the type species [1] and three known species to date [2]. It was initially classified in the family Catantopidae *sensu* Li & Xia, 2006 [3], and not assigned to a specific subfamily. Subsequently, it was placed in the subfamily Ranacridinae You et al., 1990 [4], which was later synonymized with the tribe Mesambrini Brunner von Wattenwyl, 1893, within the subfamily Catantopinae Brunner von Wattenwyl, 1893 [5,6]. Mao et al. [7] described the second species of the genus, *R. yunnanensis*. Later, the third species, *R. jinpingensis* Zheng, Lin, Deng & Shi, 2015 [8], was added to the genus. *Menglacris* Jiang & Zheng, 1994, is a genus in the subfamily Habrocneminae [2,3,9]. While *Ranacris* and *Menglacris* belong to different subfamilies in the modern classification scheme of Acrididae [2,3,10], mitogenomic and morphological evidence suggests a closer phylogenetic relationship between them [11].

When described, both *R. yunnanensis* and *R. jinpingensis* were compared with *R. albicornis* to reveal the distinguishing characters for each species. According to the original description by Mao et al. [7], *R. yunnanensis* can be distinguished from *R. albicornis* by (1) the more distinct sculptures and punctures on the body surface, especially on the dorsum of pronotum; (2) the more pronounced carinate lateral margins of vertex between eyes; (3) the nearly straight anterior and median transverse sulci of pronotum; (4) the peltate supra-anal plate of male vs. the triangular one in *R. albicornis*; (5) the distinctly smaller ratio of prozona length to metazona length in males.

The distinguishing characteristics of *R. jinpingensis* from *R. albicornis* proposed by Zheng et al. [8] are (1) the medially concave anterior margin of vertex; (2) the absent fastigial foveolae; (3) the absent furculae in males; (4) the medially triangularly protuberant posterior margin of subgenital plate in females; (5) the smaller ratio of prozona length to metazona length in males.

During the identification of specimens of *Ranacris* collected from Fenshuiling, Jinping County, Yunnan Province, China, we compared the three described species of the genus and found that *R. yunnanensis* and *R. jinpingensis* share the same type locality and exhibit no significant morphological differences.

In this study, the type specimens of all three known species of *Ranacris* were examined, and additional specimens from the type localities were analyzed to assess the morphological variation. A detailed morphological comparison was conducted to confirm whether the diagnostic characters proposed in the original references were really significantly different among species, and the taxonomic status of the *Ranacris* species was reassessed. Based on these findings, we provide a comprehensive revision of *Ranacris* and propose a new junior synonym, i.e., *Ranacris yunnanensis* Mao, Ren & Ou, 2011 = *Ranacris jinpingensis* Zheng, Lin, Deng & Shi, 2015, **syn. nov**.

## 2. Materials and Methods

### 2.1. Material Acquisition

This study is based on the specimens, including types, deposited in the insect collections of the Central South University of Forestry and Technology (CSUFT), the Biological Sciences Museum at Dali University (BMDU) and the Museum of Zoology at Shaanxi Normal University (SNU), respectively (for detailed information, see the “Material examined” section for each species). All photographs were taken using a Nikon D600 digital camera (Nikon Inc., Tokyo, Japan) or a Leica DFC 5500 system (Leica Microsystems Inc., Wetzlar, Germany), and the images were stacked using Helicon Focus version 6.0. The terminology for morphology follows Uvarov [12] and Storozhenko et al. [13].

### 2.2. Measurement Acquisition

A total of 233 specimens, including 21 of *R. albicornis,* 208 of *R. yunnanensis* and 4 of *R. jinpingensis*, were examined, among which 20 of *R. albicornis,* 42 of *R. yunnanensis* and 4 of *R. jinpingensis* were selected for morphometric analysis. Ten quantitative characters (absolute measurements) were measured according to the following criteria using a digital vernier caliper or using an ocular micrometer under a stereomicroscope. In addition, five ratio indices were computed from the measured data.

BL: body length—the distance from the apex of the fastigium to the tip of the abdomen along the longitudinal axis of the body (excluding the valvula in females).

HFL: hind femur length—the distance from the base of the hind femur to the apex along its longitudinal axis.

HFW: hind femur width—the maximum width of the hind femur along its transverse axis.

IOD: interocular distance—the minimum distance between the inner margins of the eyes.

LDE: longitudinal diameter of the eyes—the maximum length of the eyes along their longitudinal axis.

MZL: metazona length—the distance from the posterior transverse sulcus of the pronotum to the rearmost point of the posterior margin of the pronotum along the median keel. When the posterior margin of the pronotum is medially concave, MZL is measured from the midpoint of the posterior transverse sulcus to the intersection of the median keel (extended posteriorly) with the line connecting the posterolateral extremities of the posterior margin of the pronotum (excluding the median notch).

PNL: pronotum length—the distance from the frontmost point of the anterior margin of the pronotum to the rearmost point of the posterior margin along the median keel. When the anterior and posterior margins of the pronotum are medially concave, PNL is measured as the distance between the intersection points of the extension line of the median keel of the pronotum and the two tangent lines of the pronotum through the anterior and posterior margins.

PZL: prozona length—the distance from the frontmost point of the anterior margin of the pronotum to the posterior transverse sulcus of the pronotum along the median keel. When the anterior margin of the pronotum is medially concave, PZL is measured as the distance from the intersection point of the extension line of the median keel of pronotum and the connecting line between the frontmost points of the anterior margin of the pronotum to the median point of the posterior transverse sulcus of the pronotum.

SOFL: length of subocular furrow—the distance from the lower margin of the eyes to the base of the mandible along the subocular furrow.

TDE: transverse diameter of eyes—the maximum width of eyes along its transverse axis.

### 2.3. Data Analysis

The mean and standard deviation of the absolute measurements were computed using R functions “mean()” and “sd()” in the R packages of “base” and “stats”, respectively. The two-sample Wilcoxon test (also known as “Mann-Whitney” test) was performed using the R function “wilcox.test()” in the “stats” package because some variables do not follow a normal distribution. The principal components analysis (PCA) was performed using the R function “prcomp()” in the “stats” package. The plots based on the principal components (PC1, PC2 and PC3) coordinates from the PCAs were drawn using the “ggplot2” and “plotly” packages. The linear discriminant analysis was conducted using the R function “lda()” in the “MASS” package, and the score plots were drawn using the “ggplot2” package. All computations and operations were implemented in R 4.5.2 (https://www.r-project.org, accessed on 5 March 2026).

## 3. Results

### 3.1. Comparison of Qualitative Distinguishing Characters Among Ranacris Species

**Figure 1:**
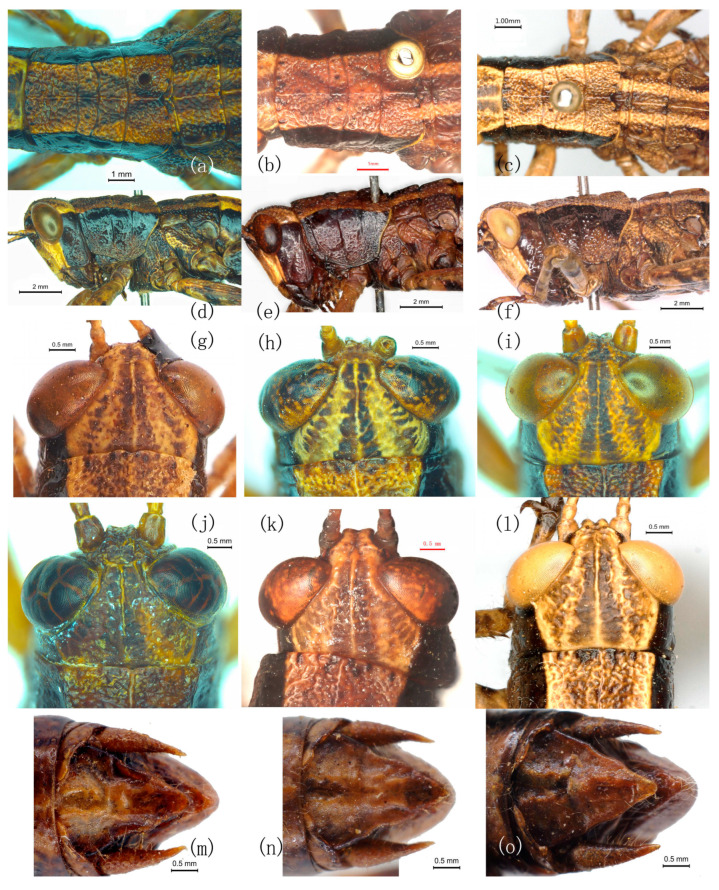
Comparison of qualitative distinguishing characters among *Ranacris* species. (**a**–**c**) Pronotum in dorsal view. (**d**–**f**) Head and pronotum in lateral view. (**g**–**l**) Vertex in dorsal view. (**m**–**o**) Supra-anal plate of male in dorsal view. (**a**,**d**,**g–j**,**m–o**) *R. albicornis*. (**g**,**m**) Holotype male of *R. albicornis*. (**n**,**o**) Paratype male of *R. albicornis*. (**b**,**e**,**k**) Holotype male of *R. yunnanensis*. (**c**,**f**,**l**) Holotype male of *R. jinpingensis*.

**Figure 2:**
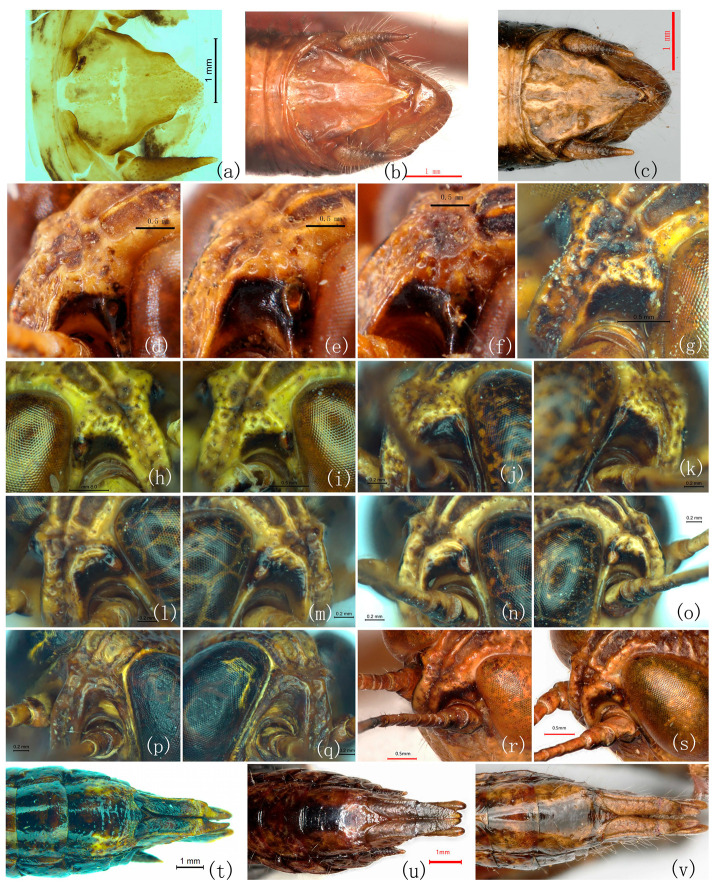
Comparison of qualitative distinguishing characteristics among *Ranacris* species (continued). (**a**–**c**) Supra-anal plate of male in dorsal view. (**d**–**s**) Fastigial foveola. (**t**–**v**) Subgenital plate of female. (**a**,**d–k**,**t**) *R. albicornis*. (**b**,**l**–**q**) *R. yunnanensis*. (**b**) Holotype male of *R. yunnanensis*. (**u**) Paratype female of *R. yunnanensis*. (**c**,**r**) Holotype male of *R. jinpingensis*. (**s**) Paratype male of *R. jinpingensis*. (**v**) Paratype female of *R. jinpingensis*.

Re-examination of the type series and additional material demonstrates that none of the diagnostic characters proposed for *R. yunnanensis* and *R. jinpingensis* are discrete; all vary continuously within populations (Table 1; Figure 1 and Figure 2).

### 3.2. Morphometric Analysis of Quantitative Characters

According to the original description, both *R. yunnanensis* and *R. jinpingensis* could be distinguished from the reference species, *R. albicornis*, by a quantitative character, the ratio of prozona length to metazona length (PZL/MZL) in male [7,8]. However, the measured data in this study (Tables S1 and S2) demonstrates that the mean of PZL/MZL in males is 2.49 ± 0.44 for *R. albicornis,* 2.39 ± 0.53 for *R. yunnanensis* and 2.23 ± 0.04 for *R. jinpingensis*, respectively (Table S3), far smaller than the values provided in the original references [1,7,8]. The females also have similar values of PZL/MZL (Table S4). The Wilcoxon test for quantitative characters demonstrates that PZL/MZL again shows no significant differences in both males and females across the three species (Table 2 and Table 3).

**Table 2:** W and *p* values resulted from Wilcoxon test of the measurements of male *Ranacris* spp.

Species Pairs	BL	PNL	HFL	HFW	IOD	LDE	TDE	SOFL
Ram–Rym	W = 47 *p* = 1.29 × 10^−4^ *	W = 196 *p* = 0.66	W = 327 *p* = 2.27 × 10^−5^ *	W = 322 *p* = 3.74 × 10^−5^ *	W = 297 *p* = 5.13 × 10^−4^ *	W = 338 *p* = 4.77 × 10^−6^ *	W = 320 *p* = 3.67 × 10^−5^ *	W = 354 *p* = 2.65 × 10^−7^ *
Ram–Rjm	W = 15 *p* = 1.00	W = 4 *p* = 0.12	W = 26 *p* = 0.13	W = 23 *p* = 0.26	W = 22 *p* = 0.33	W = 29 *p* = 0.04 *	W = 25 *p* = 0.16	W = 30 *p* = 0.03 *
Rym–Rjm	W = 43 *p* = 0.07	W = 0 *p* = 0.02 *	W = 15 *p* = 0.41	W = 12 *p* = 0.25	W = 11 *p* = 0.20	W = 19 *p* = 0.66	W = 23 *p* = 0.96	W = 38 *p* = 0.16
Species pairs	PZL	MZL	IOD/LDE	LDE/TDE	LDE/SOFL	PZL/MZL	HFL/HFW	
Ram–Rym	W = 227 *p* = 0.18	W = 135 *p* = 0.18	W = 209 *p* = 0.41	W = 213 *p* = 0.35	W = 117 *p* = 0.07	W = 239 *p* = 0.09	W = 161 *p* = 0.59	
Ram–Rjm	W = 10 *p* = 0.50	W = 2 *p* = 0.06	W = 21 *p* = 0.44	W = 17 *p* = 0.82	W = 4 *p* = 0.13	W = 26 *p* = 0.12	W = 16 *p* = 0.94	
Rym–Rjm	W = 4 *p* = 0.06	W = 4.5 *p* = 0.05	W = 15 *p* = 0.41	W = 26 *p* = 0.88	W = 15 *p* = 0.41	W = 36 *p* = 0.27	W = 29 *p* = 0.66	

Note. The acronyms for the measurements are the same as defined in the “Materials and Methods” section. The codes for the species are as follows: Ram—male *R. albicornis*, Rym—male *R. yunnanensis*, and Rjm—male *R. jinpingensis*. Asterisks indicate a significant *p*-value.

**Table 3:** W and *p* values resulted from Wilcoxon test of the measurements of female *Ranacris* spp.

Species Pairs	BL	PNL	HFL	HFW	IOD	LDE	TDE	SOFL
Raf–Ryf	W = 28 *p* = 0.22	W = 73 *p* = 0.04 *	W = 82 *p* = 0.01 *	W = 88 *p* = 1.43 × 10^−3^ *	W = 77 *p* = 0.02 *	W = 85 *p* = 0.003 *	W = 54 *p* = 0.51	W = 90 *p* = 7.60 × 10^−4^ *
Raf–Rjf	W = 8 *p* = 0.38	W = 8 *p* = 0.38	W = 10 *p* = 0.10	W = 10 *p* = 0.10	W = 8 *p* = 0.85	W = 10 *p* = 0.10	W = 8 *p* = 0.33	W = 10 *p* = 0.10
Ryf–Rjf	W = 36 *p* = 0.03 *	W = 15 *p* = 0.75	W = 25 *p* = 0.41	W = 22 *p* = 0.66	W = 7 *p* = 0.18	W = 30 *p* = 0.13	W = 18 *p* = 1.00	W = 25 *p* = 0.40
Species pairs	PZL	MZL	IOD/LDE	LDE/TDE	LDE/SOFL	PZL/MZL	HFL/HFW	
Raf–Ryf	W = 75 *p* = 0.03 *	W = 65 *p* = 0.14	W = 49 *p* = 0.79	W = 62 *p* = 0.22	W = 4 *p* = 2.44 × 10^−3^ *	W = 44 *p* = 0.97	W = 46 *p* = 0.97	
Raf–Rjf	W = 8 *p* = 0.33	W = 6 *p* = 0.86	W = 1 *p* = 0.19	W = 8 *p* = 0.38	W = 1 *p* = 0.19	W = 6 *p* = 0.86	W = 4 *p* = 0.86	
Ryf–Rjf	W = 21.5 *p* = 0.70	W = 13 *p* = 0.56	W = 6 *p* = 0.15	W = 20 *p* = 0.85	W = 17 *p* = 0.95	W = 22 *p* = 0.66	W = 19 *p* = 0.95	

Note. The acronyms for the measurements are the same as defined in the “Materials and Methods” section. The codes for the species are as follows: Raf—female *R. albicornis*, Ryf—female *R. yunnanensis*, and Rjf—female *R. jinpingensis*. Asterisks indicate a significant *p*-value.

**Figure 3:**
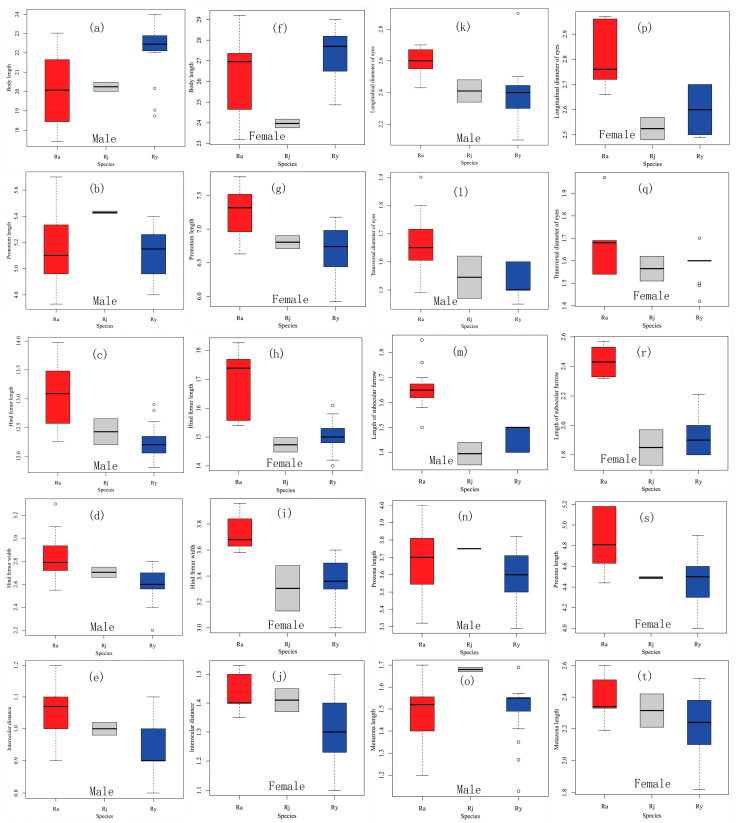
Box-and-whisker plots of ten measurements of *Ranacris* spp. (**a**–**e**,**k**–**o**) Male. (**f**–**j**,**p**–**t**) Female. (**a**,**f**) Body length. (**b**,**g**) Pronotum length. (**c**,**h**) Hind femur length. (**d**,**i**) Hind femur width. (**e**,**j**) Interocular distance. (**k**,**p**) Longitudinal diameter of eyes. (**l**,**q**) Transverse diameter of eyes. (**m**,**r**) Length of subocular furrow. (**n**,**s**) Prozona length. (**o**,**t**) Metazona length. The scale labels of the X axis for species are as follows: Ra—*R. albicornis*, Ry—*R. yunnanensis*, and Rj—*R. jinpingensis*.

Most of the measurements and ratio indices in both male and female show no significant difference between *R. albicornis* and *R. jinpingensis,* as well as between *R. yunnanensis* and *R. jinpingensis* (*p* > 0.05; Table 2 and Table 3; Figure 3), except the LDE and SOFL in males between *R. albicornis* and *R. jinpingensis* (*p* = 0.04 and 0.03, respectively; Table 2), the PNL in males (*p* = 0.02; Table 2) and the body length in females between *R. yunnanensis* and *R. jinpingensis* (*p* = 0.03; Table 3). However, seven of the ten measurements in both male and female show significant differences between *R. albicornis* and *R. yunnanensis* (*p* < 0.01 in males and *p* = 0.04, 0.01, 0.001, 0.02, 0.003, 0.0008, 0.03 in females, respectively; Table 2 and Table 3). No ratio index in males shows a significant difference between any species pair, and only LDE/SOFL in females shows a significant difference between *R. albicornis* and *R. yunnanensis* (*p* = 0.002; Table 3).

As for the distribution pattern of measured values, there is overlap in all measurements between most species pairs (Tables S1 and S2; Figure 3). The mean body length of *R. yunnanensis* in both males and females is larger than that of *R. albicornis* (Tables S3 and S4), with a significant difference in males (*p* = 0.0001; Table 2) but not in females (*p* = 0.22; Table 3). However, the extremum ranges of body length are similar between *R. albicornis* and *R. yunnanensis* and highly overlap (Tables S3 and S4; Figure 3a,f). Among the ten measurements, *R. albicornis* has six extreme ranges in males and nine in females with the largest mean. While the male *R. jinpingensis* has the largest mean pronotum length and prozona length, the male *R. albicornis* is still larger than the male *R. yunnanensis* in terms of these two values. The male *R. albicornis* has the smallest mean only in terms of the metazona length. In summary, *R. albicornis* is slightly larger than *R. yunnanensis* and *R. jinpingensis* in most measurements.

**Figure 4:**
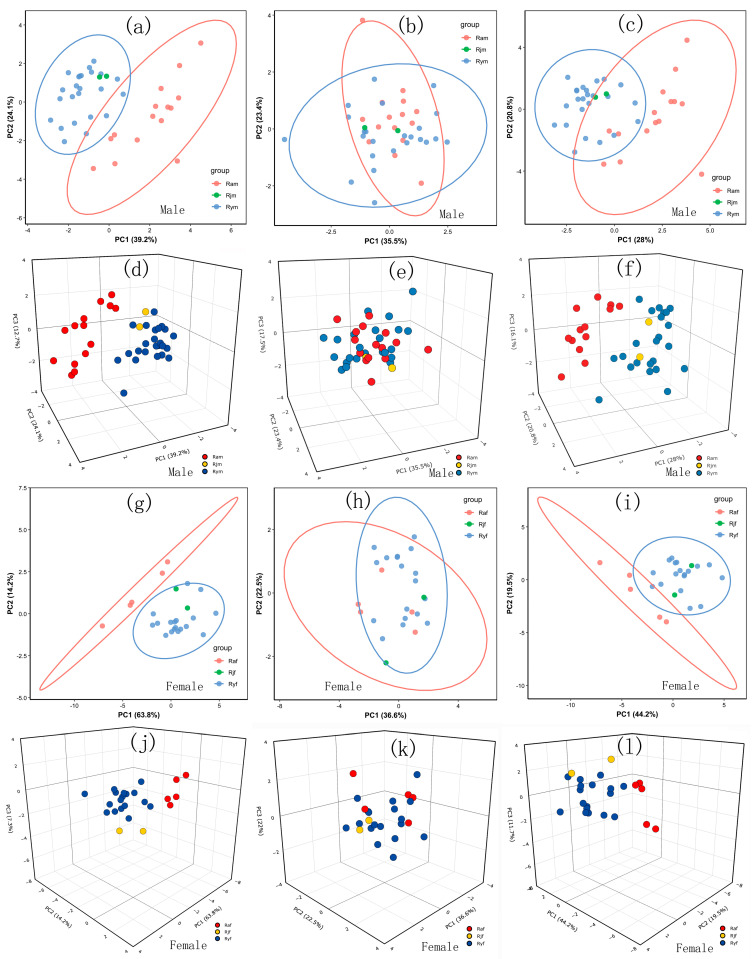
Score plots of the principal components analysis. (**a**–**f**) Male. (**g**–**l**) Female. (**a**,**d**,**g**,**j**) Score plots of PCAs based on the ten measurements. (**b**,**e**,**h**,**k**) Score plots of PCAs based on the five ratio indices. (**c**,**f**,**i**,**l**) Score plots of PCAs based on the full data. The codes for the species are as follows: Ram—male *R. albicornis*, Rym—male *R. yunnanensis*, Rjm—male *R. jinpingensis,* Raf—female *R. albicornis*, Ryf—female *R. yunnanensis*, and Rjf—female *R. jinpingensis*.

The principal components analysis (PCA) demonstrates that the first two or three principal components explain a high percentage of the variance (Figure 4), and the measurements can distinguish unambiguously between *R. albicornis* and *R. yunnanensis,* but cannot distinguish *R. jinpingensis* from *R. yunnanensis.* The two *R. jinpingensis* samples fall completely within the 95% confidence interval of *R. yunnanensis* in the PCAs based on measurements and full data (Figure 4a,c,d,f,g,i,j,l). The ratio indices have no resolution in discriminating among species (Figure 4b,e,h,k), and the combined data’s resolution is slightly reduced due to the poor performance of the ratio indices (Figure 4c,f,i,l).

**Figure 5:**
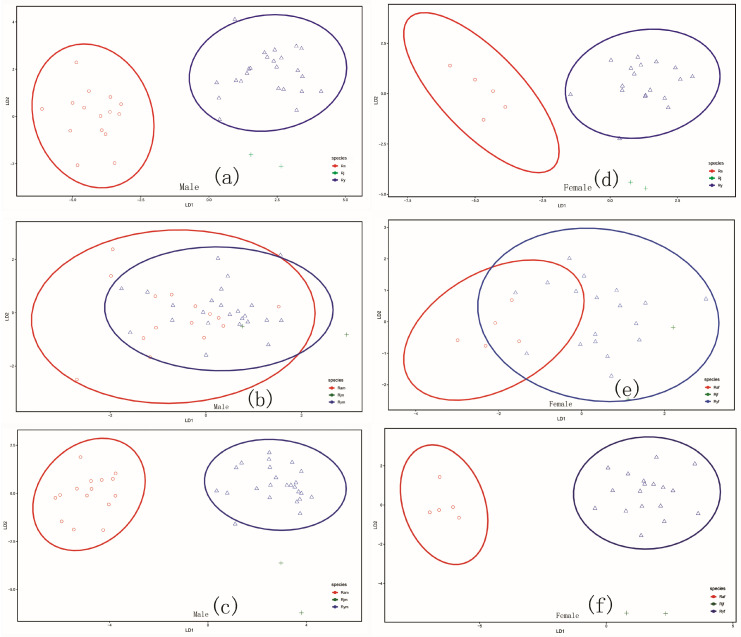
Plot of discriminant analysis of *Ranacris* spp. (**a**–**c**) Male. (**d**–**f**) Female. (**a**,**d**) Plots drawn from ten measurements. (**b**,**e**) Plots drawn from the five ratio indices. (**c**,**f**) Plots drawn from full data. The codes for the species are the same as in Figure 4.

The linear discriminant analysis (LDA) indicates that both measurements and the full data can perfectly discriminate between *R. albicornis* and *R. yunnanensis* (Figure 5a,d,c,f), but the ellipse of the 95% confidence interval for *R. jinpingensis* cannot be calculated due to too few points (Figure 5). Just as in PCAs, the ratio indices cannot accurately discriminate any species from others in LDAs again (Figure 5b,e; Table 4). Since *R. jinpingensis* differs significantly from both *R. albicornis* and *R. yunnanensis* in only a few measurements and ratio indices, we performed the LDAs again, assigning the *R. jinpingensis* samples to *R. albicornis* and *R. yunnanensis*, respectively. When the samples of *R. jinpingensis* were assigned to *R. albicornis*, one male sample was discriminated based on the measurements as *R. albicornis* with a posterior probability of 0.7857, but the other male sample was discriminated as *R. yunnanensis* with a posterior probability of 0. 5247 (Table 5 and Table S5). While the female samples of *R. jinpingensis* were all correctly discriminated as *R. albicornis* based on measurements, one female sample of *R. yunnanensis* was simultaneously incorrectly discriminated as *R. albicornis* (Table 5 and Table S6). The discrimination for both male and female based on full data was completely correct (Table 5, Tables S5 and S6). When the samples of *R. jinpingensis* were assigned to *R. yunnanensis*, the discriminations for both male and female based on measurement and full data were all correct (Table 6, Table S7 and S8). The proportion of incorrect discrimination was always very high based on ratio indices (Table 5 and Table 6).

**Table 4:** Contingency table of the counts of samples of *Ranacris* spp. at each combination of the assigned species and the discriminated species.

Species	Male									Female								
	Discrimination Based on Measurements			Discrimination Based on Ratio Indices			Discrimination Based on Full Data			Discrimination Based on Measurements			Discrimination Based on Ratio Indices			Discrimination Based on Full Data		
	Ram	Rym	Rjm	Ram	Rym	Rjm	Ram	Rym	Rjm	Ram	Rym	Rjm	Ram	Rym	Rjm	Ram	Rym	Rjm
Ram	15	0	0	7	4	0	15	0	0	5	0	0	5	2	0	5	0	0
Rym	0	0	2	0	0	1	0	0	2	0	0	2	0	0	1	0	0	2
Rjm	0	24	0	8	20	1	0	24	0	0	18	0	0	16	1	0	18	0

Note. The species names used as column names represent the assigned species of the samples, and the row names are referred to as the discriminated species of the samples. The number in each cell of the table indicates the frequency of samples of a given assigned species being identified as a given discriminated species.

**Table 5:** Contingency table of the counts of samples at each combination of the assigned species and the discriminated species when the samples of *R. jinpingensis* were assigned to *R. albicornis*.

Species	Male						Female					
	Discrimination Based on Measurements		Discrimination Based on Ratio Indices		Discrimination based on Full Data		Discrimination Based on Measurements		Discrimination Based on Ratio Indices		Discrimination Based on Full Data	
	Ram	Rym	Ram	Rym	Ram	Rym	Raf	Ryf	Raf	Ryf	Raf	Ryf
Ram	16	0	5	4	17	0	7	1	4	1	7	0
Rym	1	24	12	20	0	24	0	17	3	17	0	18

**Table 6:** Contingency table of the counts of samples at each combination of the assigned species and the discriminated species when the sample of *R. jinpingensis* was assigned to *R. yunnanensis*.

Species	Male						Female					
	Discrimination Based on Measurements		Discrimination Based on Ratio Indices		Discrimination Based on Full Data		Discrimination Based on Measurements		Discrimination Based on Ratio Indices		Discrimination Based on Full Data	
	Ram	Rym	Ram	Rym	Ram	Rym	Raf	Ryf	Raf	Ryf	Raf	Ryf
Ram	15	0	7	4	15	0	5	0	5	2	5	0
Rym	0	26	8	22	0	26	0	20	0	18	0	20

### 3.3. Taxonomy

#### 3.3.1. Genus *Ranacris* You & Lin, 1983

*Ranacris* You & Lin, 1983: 257 [1]; Otte, 1995: 334 [10]; Yin et al., 1996: 608 [14]; Jiang & Zheng, 1998: 188 [15]; Li & Xia, 2006: 637 [3]; Mao et al., 2011: 152 [7]; Zheng et al., 2015: 177 [8]; Storozhenko, 2018: 56 [6]; Zhang et al., 2023: 18 [11].

**Type species**: *Ranacris albicornis* You & Lin, 1983, by original designation.

**Generic diagnosis.** Body medium-sized. Head shorter than pronotum, face distinctly reclinate in profile. Antennae filiform, reaching or exceeding the posterior margin of pronotum. Pronotum tectiform, with slightly raised median keel and distinct lateral keels. Prosternal spine conical and straight; apex pointed. Lateral lobes of meso- and metasterna broadly separated throughout. Tegmen vestigial, hind wing absent. Hind femora with upper basal lobe of outer surface distinct longer than lower one, upper median keel sparsely serrate and denticulate apically, and lower genicular lobe rounded. Hind tarsi normal, arolium developed. Tympanal organ large and nearly rounded. In males, the tenth abdominal tergite broadly split in the middle, with a small arciform furcula at each side of the split; supra-anal plate triangular; subgenital plate short, conical, with apex acute and tilted upwards. Cerci conical. Upper valvula straight.

**Distribution.** China (Guangxi, Yunnan).

#### 3.3.2. Species. *Ranacris albicornis* You & Lin, 1983

**Figure 6:**
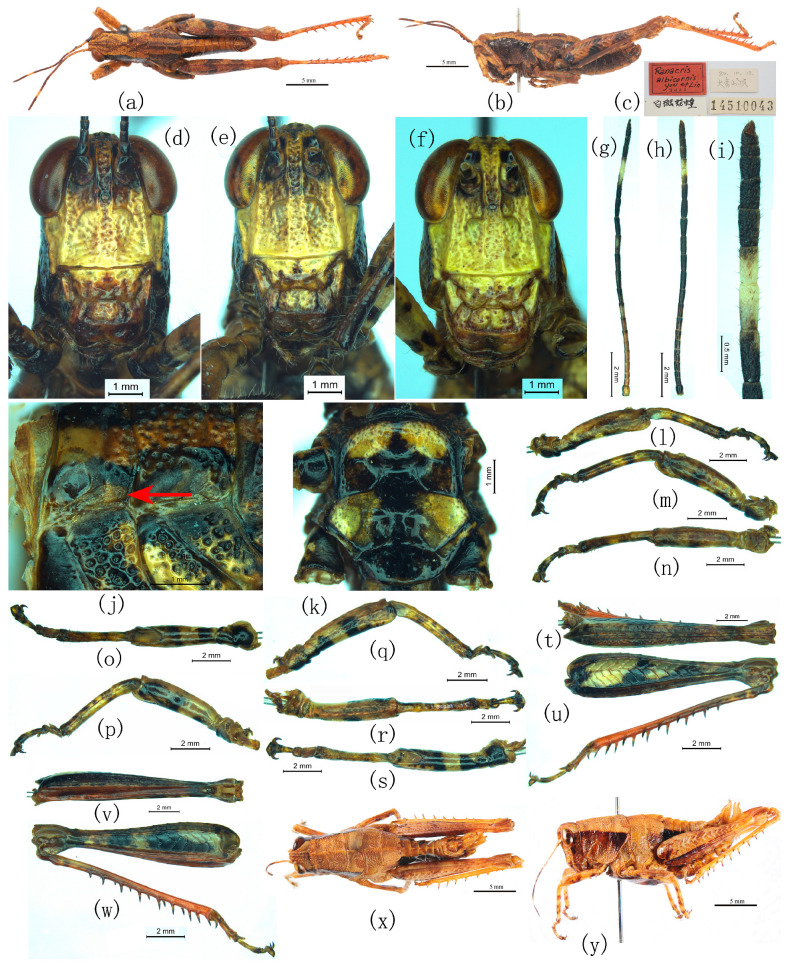
Habitus and closeups of *Ranacris albicornis* You & Lin, 1983. (**a**–**c**) Holotype male. (**a**,**b**) In dorsal and lateral views. (**c**) Labels (The Chinese in the lower left is the Chinese name of *R. albicornis*, and that in the upper right is the detailed type locality). (**d**–**w**) Closeups of male. (**d**–**f**) Frons. (**g**–**i**) Antenna. (**g**,**h**) In dorsal and lateral views. (**i**) Apex. (**j**) Left tegmen. (**k**) Meso- and metasterna. (**l**–**o**) Fore leg in inner, outer, dorsal and ventral views. (**p**–**s**) Middle leg in outer, inner, dorsal and ventral views. (**t**–**w**) Hind femur in dorsal, outer, ventral and inner views. (**x**,**y**) Female in dorsal and lateral views.

**Figure 7:**
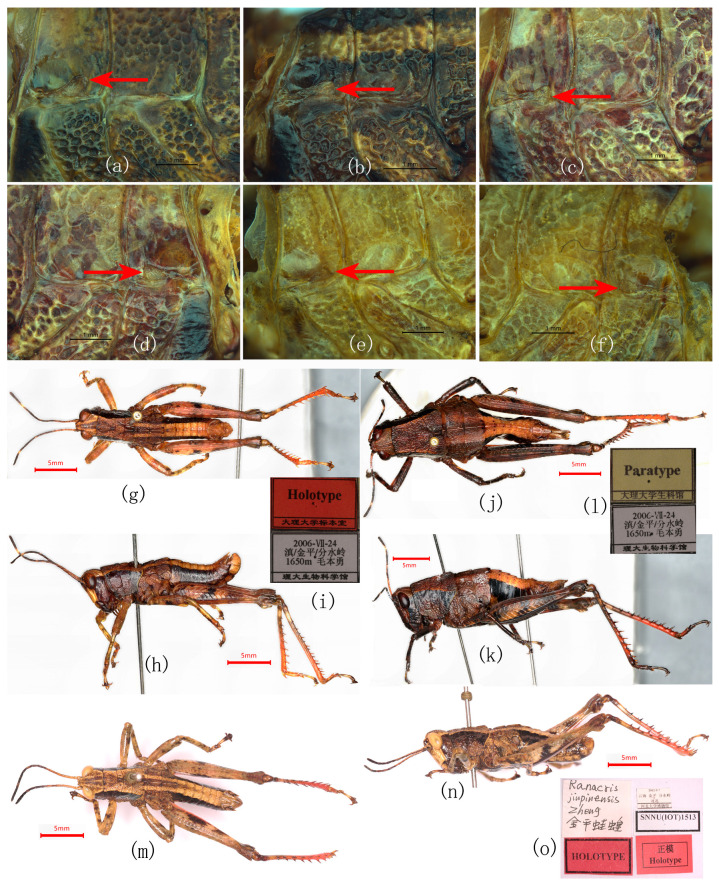
Habitus and closeups of *Ranacris* spp. (**a**) Left tegmen of female *R. albicornis*. (**b**) Left tegmen of male *R. yunnanensis*. (**c**–**f**) Tegmen of female *R. yunnanensis*. (**g**–**l**) Types of *R. yunnanensis*. (**g**,**h**) Holotype male in dorsal and lateral view. (**i**) Labels (The Chinese in the lower label is the detailed type locality and the name of the collector). (**j**,**k**) Paratype female in dorsal and lateral view. (**l**) Labels (The Chinese in the lower lable is the detailed type locality and the name of the collector). (**m**–**o**) Types of *R. jinpingensis*. (**m**,**n**) Holotype male in dorsal and lateral views. (**o**) Labels (The Chinese in the upper left is the Chinese name of *R. jinpingensis*, and that in the upper right is the detailed type locality).

**Figure 8:**
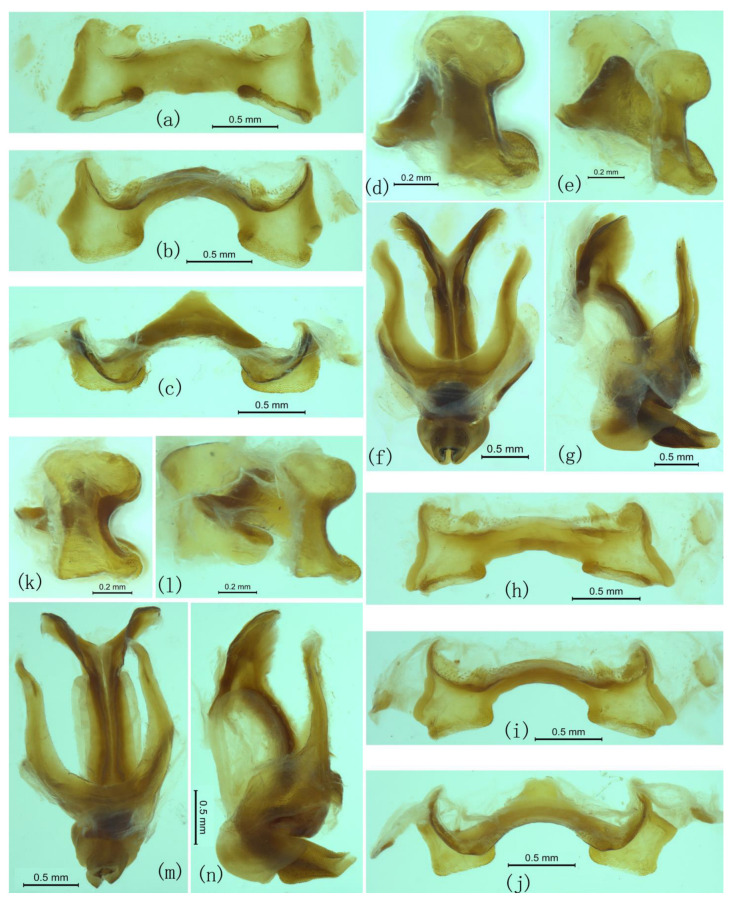
Male genitaliae of *Ranacris albicornis* and *R. yunnanensis*. (**a**–**g**) *R. albicornis*. (**a**–**e**) Epiphallus in dorsal, dorsofrontal, frontal, lateral and ventrolateral views. (**f**,**g**) Phallic complex in dorsal and lateral views. (**h**–**n**) *R. yunnanensis*. (**h**–**l**) Epiphallus in dorsal, dorsofrontal, frontal, lateral and ventrolateral views. (**m**,**n**) Phallic complex in dorsal and lateral views.

(Figure 6, Figure 7a and Figure 8a–g)

*Ranacris albicornis* You & Lin, 1983 [1]: 258; You et al., 1990: 70 [4]; Otte, 1995: 334 [10]; Jiang & Zheng, 1998: 189 [15]; Li & Xia, 2006: 638 [3]; Mao et al., 2011: 152, 154 [7]; Zheng et al., 2015: 179 [8]; Zhang et al., 2023: 7, 19, 21 [11].

**Type locality:** China (Pingxiang, Guangxi).

**Material examined.** Holotype ♂, the peak of Daqingshan, Pingxiang County, Guangxi, China, 1000 m, 13 October 1980, leg. Rizhao Lin, Hongxian Ji, Qijing You (SEMCAS, 14510043); paratypes, 2♂, data the same as holotype (SEMCAS, 14510044, 14510045). **Other material examined**: 8♂, 2♀, Diding Nature Reserve, Nanpo Township, Jingxi County, Guangxi, China, 800–900 m, 8 August 2010, leg. Jianhua Huang; 5♂, 3♀, Diding Nature Reserve, Nanpo Township, Jingxi County, Guangxi, China, 23.1207806° N, 105.9738116° E, 20 August 2025, leg. Zhongke Lv.

**Morphology.**

**Male.** Body medium-sized, well proportioned. Head shorter than pronotum, as long as or slightly longer than half of the pronotum length. Vertex slightly convex in lateral view, distinctly longitudinally carinated, either thickly or finely, along the lateral margins between eyes, and transversely carinated slightly before the narrowest part between eyes, with a distinct fine median keel which becomes obsolete on the hind half; fastigium hexagonal, strongly reclined forwards with anterior margin distinctly concave; foveola small, triangular (Figure 2h), or rectangular (Figure 2f,g), or indistinct (Figure 2d,e,i), or even nearly absent (Figure 2j,k). Antennae filiform, 25-segmented, distinctly exceeding the posterior margin of pronotum. Frons broad and flat, with a rounded fine sulcus at each side of the frontal ridge below the antennal socket and an oval prominence between the sulcus and the antennal socket. Frontal ridge deeply and broadly sulcate nearly throughout, lateral margins distinct, straight and parallel. Facial keels distinct and nearly straight. Clypeus inversely trapezoidal, with lateral margins slightly concave or bearing a small tubercle in the middle, anterior margin broadly and triangularly emarginate (Figure 6d–f). Eyes elliptical, with vertical diameter about 1.3–1.7 times the horizontal diameter and about 1.4–1.7 times the length of the subocular furrow. Subocular furrow broadly depressed. Genae with a distinct and straight vertical carina at or slightly behind the extension line of the longitudinal axis of eyes (Figure 1d). Pronotum tectiform, densely and coarsely punctured, with anterior margin slightly narrower than posterior margin and nearly straight (Figure 1a) or sometimes minutely concave in the middle (Figure 1g,h); lateral keels distinct, slightly incurved at the anterior transverse sulcus; median keel distinct throughout; all of the three transverse sulci distinct and interrupting the median keel, with the anterior and posterior ones straight and the median one posteriorly circular-arc-shaped; posterior transverse sulcus situated far behind the middle of pronotum, and prozona length about 2.10–3.08 times of metazona length. Meso-, metanotal and abdominal tergites distinctly carinate medially, with the meso-, metanotal and the first two abdominal tergites raised tectiformly. Interspace of the lateral lobes of mesosternum trapezoidal, with its maximum width about 1.77 times its length. Interspace of the lateral lobes of metasternum inversely trapezoidal, with its width distinctly narrower than that of its lateral lobes. Tegmina vestigial and hind wing absent. Hind femora well-proportioned and distinctly exceeding the apex of abdomen, with its length approximately 4.00–5.02 times its maximum width. Arolium developed, nearly as long as the length of claw. Supra-anal plate triangular, much longer than broad, with lateral margins bisinuate and elevated in the basal third; sometimes the lateral margins deeply emarginate subapically to form a mucronate apex (Figure 1m–o and Figure 2a); dorsum broadly longitudinally sulcated throughout and interrupted by a transverse carina and raised area. Cerci long, conical, broad and slightly compressed basally, covered with long pubescence; apex sharply pointed.

Epiphallus not divided into two symmetric halves; lateral plates broad, with lateral margins slightly oblique inwards and concave in the middle; anterior projections large, with bluntly rounded apices; ancorae small and triangular, slightly curved ventrally with apices pointed; posterior projections short and conical; bridge moderately broad, with anterior and posterior margins nearly straight in dorsal view, and posterior half strongly and crateriformly depressed in the middle in frontal and lateral views (Figure 8d,e); lophi large and rectangular in frontal view, obliquely situated along the inner margins of lateral plate; oval sclerites present. Phallic complex robust; basal valves of penis large and broad; zygoma broad; apodemes moderately long and robust, not reaching the apices of the basal valves of penis; valves of cingulum and apical valves of penis upcurved and conical with pointed apex in lateral view.

**Female**. Body larger than male, slightly plump. Eyes with vertical diameter about 1.50–1.77 times the horizontal diameter and about 1.04–1.27 times the length of subocular furrow. Prozona length about 2 times the metazona length. Interspace of the lateral lobes of mesosternum with its maximum width 1.58 times its minimum width and twice its length. Hind femora length approximately 4.37–4.42 times its width. Ovipositor moderately long; dorsal valvulae slightly robust, with apex strongly narrowed, sharply pointed and slightly curved upward; dorsolateral margins of the dorsal valvulae densely denticulate; ventral valvulae slightly slender, broad basally, gradually narrowed apically, with apex sharply pointed and slightly curved downwards. Subgenital plate quadrangular, with posterior margin triangularly protruding in the middle.

**Coloration.**

Body castaneous to buffy. Labrum and clypeus brown to yellowish brown. Frons buffy below median ocellus, and dark brown (Figure 6d) to yellowish brown (Figure 6e, f) above median ocellus. Antennae black to dark brown, with a distinct pale annulation near the apex (Figure 6g–i). Vertex with a broad long-trapezoidal dark brown stripe in the middle. Genae black to blackish brown behind the posterior margin of the subocular furrow. Pronotal dorsum with an indistinct V-shaped dark brown stripe both before and behind the median transverse sulcus; postocular band black. Meso-, metanotum and the first abdominal tergite with a broad and black longitudinal stripe in the middle and at each side, respectively; mesosternum black with a pair of large oval yellowish brown maculations at the anterior half (Figure 6k). Fore and middle legs buffy with some distinct or indistinct black transverse bands. Hind femora dark brown; outer surface with three distinct and one indistinct transverse black maculations (Figure 6u) and dorsal surface with four indistinct transverse dark maculations at the corresponding positions (Figure 6t); ventral surface with outer half black variegated with yellowish brown and inner half reddish brown. Hind tibiae reddish, basal half yellowish to dark brown with a distinct or indistinct yellowish brown annulation near the base. Abdomen black on the lateral surface; sternum has broad longitudinal black stripe in the middle.

**Measurements.** Body length: ♂, 17.40–23.03 mm, ♀, 23.18–29.21 mm; pronotum length: ♂, 4.70–5.70 mm, ♀, 6.50–7.78 mm; tegmen length: ♂, 1.18–1.24 mm, ♀, 1.21–1.33 mm; hind femur length: ♂, 12.25–13.98 mm, ♀, 15.39–18.26 mm.

**Distribution.** China (Guangxi).

#### 3.3.3. *Ranacris yunnanensis* Mao, Ren & Ou, 2011

**Figure 9:**
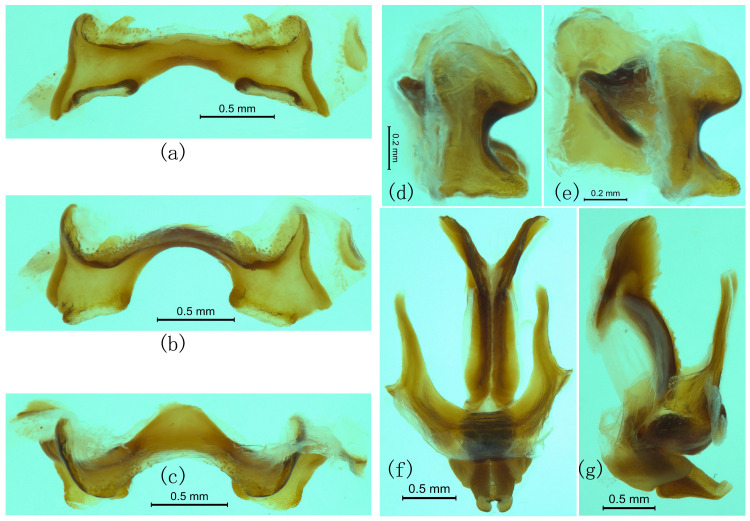
Male genitalia of *Ranacris jinpingensis*. (**a**–**e**) Epiphallus in dorsal, dorsofrontal, frontal, lateral and ventrolateral views. (**f**,**g**) Phallic complex in dorsal and lateral views.

(Figure 7b–o, Figure 8h–n and Figure 9).

*Ranacris yunnanensis* Mao, Ren & Ou, 2011: 305 [7].

*Ranacris jinpingensis* Zheng, Lin, Deng & Shi, 2015: 178 [8]. **Syn. nov**.

**Type locality:** China (Fenshuiling Nature Reserve, Jinping County, Yunnan).

**Material examined. *Ranacris yunnanensis***: Holotype ♂, Fenshuiling Nature Reserve, Jinping County, Yunnan Province, China, 1650 m, 24 July 2006, leg. Benyong Mao; paratypes: 59♂, 115♀, data the same as holotype except a larger altitude range of 1650–1850 m and more than one collectors: Benyong Mao, Zizhong Yang, Jishan Xu, Haoyu Liu, Qiqi Wu, and Juntong Lang (BMDU except 2♂, 2♀ in SEMCAS); fifteen 15♂, 13♀, data the same as paratypes (BMDU); 1♂, Fenshuiling Nature Reserve, JinPing County, Yunnan, China, 103.222° E, 22.854° N, 14 September 2024, leg. Ting Luo, Yanting Qin; 2♂, 2♀, Fenshuiling, JinPing County, Yunnan, China, 103.222° E, 22.854° N, 17 September 2024, leg. Ting Luo, Yanting Qin (CSUFT). ***Ranacris jinpingensis*:** holotype ♂, Fenshuiling, JinPing County, Yunnan Province, China, 7 September 2012, leg. Xun Bian; paratypes: 1♂, 2♀, data the same as holotype (SNU).

**Morphology.**

This species has no significant difference from *R. albicornis* in all qualitative characters, except the body size of *R. albicornis* is slightly larger than that of *R. yunnanensis*. However, the body length of *R. albicornis* measured in this study is slightly smaller than that of *R. yunnanensis* (Tables S1–S4). Epiphallus with the apex of the funnelform depression of bridge directed upwards (Figure 8k,l). There is no significant difference between *R. yunnanensis* and *R. jinpingensis*, including the male genitalia (Figure 8h–n and Figure 9).

**Measurements.** Body length: ♂, 18.60–24.00 mm, ♀, 23.20–29.00 mm; pronotum length: ♂, 4.80–5.44 mm, ♀, 5.92–7.18 mm; tegmen length: ♂, 0.90–1.04 mm, ♀, 1.33–1.41 mm; hind femur length: ♂, 11.50–12.90 mm, ♀, 14.00–16.10 mm.

**Distribution.** China (Jinping, Yunnan).

**Remarks.** The type localities of *R. yunnanensis* and *R. jinpingensis* are the same place, Fenshuiling Nature Reserve, Jinping County, Yunnan Province, China. These two species show no significant differences in qualitative characters, as well as in nearly all measurements and ratio indices, according to the results of the morphometric analysis. Therefore, we treated *R. jinpingensis* as a junior synonym of *R. yunnanensis* herein.

## 4. Discussion

Body size is associated with many aspects of an organism’s biology, including local adaptations to different climatic conditions, female fecundity and male mating success, and so on [16]. Generally, the increase in altitude constrains growth rates and development times, leading to a smaller adult body size at high elevations than in lowlands [17,18,19,20,21]. *R. albicornis* inhabits altitudes between 700 and 1000 m, and *R. yunnanensis* occurs at high elevations between 1600 and 1950 m. Therefore, the larger body size of *R. albicornis* than *R. yunnanensis* can be explained at least partially by the hypothesis of “local adaptations to different climatic conditions” [16].

Usually, the body length of grasshoppers correlates positively with body size, i.e., a species with a larger body size generally has a larger body length correspondingly. However, in this study, as mentioned in the Results section, *R. albicornis* shows a larger body size but a smaller body length than *R. yunnanensis* in both males and females. How does this inconsistency occur? As far as we know, at least three factors may influence the measured value of body length: the accurate recognition of mature nymphs, the preserving method of specimens and the biological attribute of abdomen in female grasshoppers. (1) *Ranacris* species have vestigial tegmina completely covered by the lateral lobe of pronotum (Figure 6j), leading to difficulty in accurately discriminating between adult and mature nymph. While the sclerotization of integument and the development of genitalia can be used to help determine the adulthood of the samples, it is still difficult to completely ensure the accuracy of the determination because there is no explicit standard for the maturity of the integument and genitalia, and the determination is mainly based on personal experience. If a slightly high proportion of mature (last-instar) nymphs are wrongly recognized as adults and included in the measured samples, the measured values will decrease to some extent. (2) Some measured samples of *R. albicornis* collected in 2010 are shrunken strongly, especially the abdomen, due to dehydration when preserved in absolute ethyl alcohol, and these individuals generally exhibit smaller measured values, suggesting that these individuals may be mature nymphs and probably result in a substantial decrease in body length in *R. albicornis*. Dehydration with absolute ethyl alcohol will not only substantially decrease the body length of nymphs but also slightly shrink that of adults. For the purpose of preserving molecular material and the convenience of preventing the specimens from decaying in the field, all specimens of *R. albicornis* and the types of *R. jinpingensis* were preserved in absolute ethyl alcohol. However, the measured samples of *R. yunnanensis*, including partial types, were all poisoned via ethyl acetate in the field and dried naturally at ordinary temperatures, and this treatment has a much smaller influence on the body shrinkage. Since dehydration via absolute ethyl alcohol mainly acts on the highly retractile abdomen, body length becomes the most affected measurement, resulting in a smaller body length in *R. albicornis* than in *R. yunnanensis*. (3) The abdomen of female grasshoppers is biologically linked to the reproductive state (e.g., abdomen extension for oviposition). Individuals in the period of oviposition exhibit a much longer body length than in normal conditions. According to our observations, the time of collecting the examined specimens of *R. albicornis* may be during the early stages of eclosion, whereas the large number of recorded types of *R. yunnanensis* indicates it was at the peak of reproduction [7]. Of course, there is another possibility that the body length of *R. yunnanensis* may be larger than that of *R. albicornis*, but this requires further confirmation. Regardless, the total body length should be used carefully or even ignored sometimes as a diagnostic character, especially for closely related species, due to its substantial intraspecific variation [22].

When *R. yunnanensis* and *R. jinpingensis* were described, the authors indicated that male *R. albicornis* had a significantly larger ratio value of PZL/MZL than *R. yunnanensis* and *R. jinpingensis.* However, the remeasured values of PZL/MZL from the holotype male and two paratype males of *R. albicornis* show that it is significantly smaller (only 2.6–3.1; Table S1) than that (3.5–3.7) presented by You & Lin [1] in the original reference. In addition, the means of PZL/MZL of *R. yunnanensis* and *R. jinpingensis* in males are also significantly smaller (Table S3) than those presented in the original reference [7,8]. No significant difference in PZL/MZL was detected between any species pair in both male and female (Tables S3 and S4). Why did such an inconsistency occur? Besides inherent errors from different measurers, the use of different definitions or criteria for measurements may be another important factor contributing to such inconsistency. There are different definitions for a few absolute measurements of body parts. For example, Uvarov [12] defined the body length as “from apex of head to tip of abdomen”, but Mao et al. [7] and Hill [23] defined it as “dorsally from the fastigium vertices to the distal end of the genicular lobe of caudal femur in a parallel plane with the abdomen”. Hochkirch [22] illustrated the criteria for 27 measurements (excluding body length due to its sensitivity to shrinkage) and defined the metazona length in the figure as “the length from posterior transverse sulcus to the end of the median keel of pronotum”, and this was followed by Mao et al. [7]. While there is no clear definition for any measurement in the representative monographs of grasshopper taxonomy in China [3,24,25,26,27,28], most Chinese acridologists understand the metazona length as “the distance from the posterior transverse sulcus of pronotum to the rearmost point of the posterior margin of pronotum along the median keel” and accept Uvarov’s definition of body length [12]. Hochkirch’s definition of metazona length [22] means a smaller measured value, and this will result in a larger ratio value of PZL/MZL. Therefore, it is extremely important for all acridologists to use a unified set of criteria for the absolute measurements. The body length should be defined as the distance from one point of the soma to another one but not that of the appendages. We think Mao et al.’s [7] and Hill’s [23] definitions do not meet the essential implication for body length. Similarly, the metazona length should include the most distant point but not the bottom of the notch when the posterior margin of the pronotum is concave in the middle, and the pronotum length should equal the sum of prozona length and metazona length.

While *Ranacris* was initially described as apterous [1], this study demonstrates that *Ranacris* species have vestigial tegmina (Figure 6j and Figure 7a–f), and this finding further confirms the close relationship between *Ranacris* and *Menglacris* revealed by molecular evidence in a previous study [11]. Because the sole morphological diagnosis of aptery previously used to define *Ranacris* no longer exists and the degree of reduction in tegmen from micropterous to apterous condition is not satisfactory for generic distinction in some groups due to the substantial variation even within species [29,30,31], it is reasonable to place the two genera within the same subfamily or tribe, or even to merge *Ranacris* and *Menglacris* into one genus after a comprehensive revision.

## Figures and Tables

**Table 1:** Comparison table of the original diagnostic characters proposed for *R. yunnanensis* and *R. jinpingensis* and the observation variation in this study.

Character	Original Diagnosis	Observed Variation	Taxonomic Value
Integumental punctures	Distinct [7] vs. undescribed [1,8].	The dorsum of pronotum is densely and coarsely punctured in all species (Figure 1a–c). The punctures are slightly sparser on the dorsum of head (Figure 1g–i). Lateral lobe of pronotum is smooth and shining on the upper half, with a few punctures on the lower half (Figure 1d–f).	Invalid
Lateral margins of vertex	Carinate [7] vs. undescribed [1,8].	Distinctly carinate in all species, either thick (Figure 1g,k,l) or fine (Figure 1h–j).	Invalid
The shape of the transverse sulci of pronotum	Anterior and median transverse sulci of pronotum nearly straight [7] vs. undescribed [1,8].	Anterior and posterior transverse sulci straight and median transverse sulcus posteriorly circular arc in all species (Figure 1a–c).	Invalid
The shape of male supra-anal plate	Long shield-shaped with lateral margins slightly concave near apex, posterior margin distinctly constricted and protruded lingulately in the middle [7] vs. triangular [1] or long triangular [8].	Triangular with some extent of variation among individuals; no significant difference found among the three species (Figure 1m–o and Figure 2a–c).	Invalid
Anterior margin of vertex	Concave medially [7,8] vs. rounded [1].	Concave medially in all species (Figure 1g–l).	Invalid
Foveola	Triangular [1] vs. indistinct [7] or absent [8].	Continuous in all species (Figure 2d–s).	Invalid
Furculae in males	Small and triangular [1] vs. absent [7,8].	Arciform in the male of all species (Figure 1m–o and Figure 2a–c).	Invalid
Posterior margin of subgenital plate in females	Slightly concave medially [1] vs. nearly straight [7] or triangularly protuberant medially [8].	Triangularly protuberant medially in all species (Figure 2t–v).	Invalid

## Data Availability

Original data of measurements are available in Tables S1 and S2.

## Supplementary Materials

The following supporting information can be downloaded at: https://www.mdpi.com/article/10.3390/insects17030298/s1, where Tables S1–S8 are published along with the manuscript. Table S1. Measurements of male *Ranacris* spp. Table S2. Measurements of female *Ranacris* spp. Table S3. Statistics of the measurements and indices of male *Ranacris* spp. Table S4. Statistics of the measurements and indices of female Ranacris spp. Table S5. Results of discriminant analysis of male *Ranacris* spp. when the sample of *R. jinpingensis* was assigned to *R. albicornis*. Table S6. Results of discriminant analysis of female *Ranacris* spp. when the sample of *R. jinpingensis* was assigned to *R. albicornis*. Table S7. Results of discriminant analysis of male *Ranacris* spp. when the sample of *R. jinpingensis* was assigned to *R. yunnanensis*. Table S8. Results of discriminant analysis of female *Ranacris* spp. when the sample of *R. jinpingensis* was assigned to *R. yunnanensis*.
