# Supplementary material for: Revision of the Genus Ranacris You & Lin, 1983 (Orthoptera: Acrididae: Catantopinae), with Proposal of a New Synonym
Source: Insects. 2026 Mar 9;17(3):298. doi: 10.3390/insects17030298 (PMC13026737; doi:10.3390/insects17030298)
Supplement: Supplementary file 1 [file insects-17-00298-s001.zip › insects-4158921-supplementary.pdf]

Table S1. Measurements of male *Ranacris* spp.

Sample code	BL	PNL	HFL	HFW	IOD	LDE	TDE	SOFL	PZL	MZL	IOD/ LDE	LDE/ TDE	LDE/ SOFL	PZL/ MZL	HFL/ HFW
Rahm	21.70	5.10	13.60	3.00	1.00	2.50	1.90	1.50	3.70	1.40	0.4000	1.3158	1.6667	2.6429	4.5333
Rapm_01	21.60	4.90	13.20	3.30	1.20	2.70	1.60	1.70	3.70	1.20	0.4444	1.6875	1.5882	3.0833	4.0000
Rapm_02	21.60	5.10	13.30	3.10	0.90	2.70	1.80	1.60	3.70	1.40	0.3333	1.5000	1.6875	2.6429	4.2903
Ram_01	22.02	5.70	13.98	2.78	0.90	2.61	1.78	1.85	4.00	1.70	0.3448	1.4663	1.4108	2.3529	5.0288
Ram_02	20.83	4.89	12.98	2.64	1.08	2.60	1.67	1.67	3.58	1.31	0.4154	1.5569	1.5569	2.7328	4.9167
Ram_03	20.07	5.21	13.05	2.76	1.18	2.59	1.74	1.64	3.73	1.48	0.4556	1.4885	1.5793	2.5203	4.7283
Ram_04	23.03	5.47	13.96	2.85	0.95	2.61	1.65	1.76	3.89	1.58	0.3640	1.5818	1.4830	2.4620	4.8982
Ram_05	21.99	5.32	13.38	2.79	1.00	2.68	1.65	1.65	3.81	1.51	0.3731	1.6242	1.6242	2.5232	4.7957
Ram_06	18.96	5.24	13.09	2.96	1.07	2.59	1.63	1.58	3.72	1.52	0.4131	1.5890	1.6392	2.4474	4.4223
Ram_07	17.41	4.73	12.41	2.72	1.07	2.43	1.49	1.61	3.33	1.40	0.4403	1.6309	1.5093	2.3786	4.5625
Ram_08	19.79	5.35	12.73	2.71	1.13	2.66	1.66	1.66	3.81	1.54	0.4248	1.6024	1.6024	2.4740	4.6974
Ram_09	17.57	5.02	12.27	2.55	1.08	2.56	1.61	1.63	3.45	1.57	0.4219	1.5901	1.5706	2.1975	4.8118
Ram_10	17.92	5.04	12.25	2.72	1.03	2.51	1.58	1.65	3.51	1.53	0.4104	1.5886	1.5212	2.2941	4.5037
Ram_11	19.93	5.40	13.58	2.91	1.09	2.70	1.69	1.63	3.82	1.58	0.4037	1.5976	1.6564	2.4177	4.6667
Ram_12	17.90	4.84	12.38	2.87	1.11	2.54	1.54	1.68	3.32	1.52	0.4370	1.6494	1.5119	2.1842	4.3136
Ryhm	23.00	5.15	12.80	2.70	0.85	2.10	1.50	1.50	3.60	1.55	0.4048	1.4000	1.4000	2.3226	4.7407
Rypm_01	22.20	5.26	12.20	2.20	0.95	2.40	1.50	1.40	3.71	1.55	0.3958	1.6000	1.7143	2.3935	5.5455
Rypm_02	22.80	5.26	12.40	2.50	0.90	2.50	1.60	1.50	3.71	1.55	0.3600	1.5625	1.6667	2.3935	4.9600
Rypm_03	22.80	5.40	12.20	2.60	1.00	2.30	1.50	1.50	3.71	1.69	0.4348	1.5333	1.5333	2.1953	4.6923
Rypm_04	23.00	4.91	12.10	2.60	1.10	2.40	1.50	1.40	3.50	1.41	0.4583	1.6000	1.7143	2.4823	4.6538
Rypm_05	23.20	5.15	12.30	2.80	0.90	2.90	1.60	1.50	3.60	1.55	0.3103	1.8125	1.9333	2.3226	4.3929
Rym_01	22.50	5.15	12.10	2.60	0.90	2.30	1.50	1.50	3.60	1.55	0.3913	1.5333	1.5333	2.3226	4.6538
Rym_02	22.20	4.84	12.00	2.50	1.00	2.30	1.50	1.40	3.29	1.55	0.4348	1.5333	1.6429	2.1226	4.8000
Rym_03	24.00	4.84	12.60	2.70	0.90	2.50	1.60	1.50	3.71	1.13	0.3600	1.5625	1.6667	3.2832	4.6667
Rym_04	22.50	4.98	12.00	2.70	1.00	2.40	1.50	1.50	3.71	1.27	0.4167	1.6000	1.6000	2.9213	4.4444
Rym_05	22.10	5.15	12.20	2.70	0.90	2.40	1.50	1.40	3.60	1.55	0.3750	1.6000	1.7143	2.3226	4.5185
Rym_06	22.40	5.05	12.10	2.70	0.90	2.40	1.50	1.40	3.50	1.55	0.3750	1.6000	1.7143	2.2581	4.4815
Rym_07	23.30	4.94	12.30	2.60	0.90	2.30	1.50	1.40	3.39	1.55	0.3913	1.5333	1.6429	2.1871	4.7308
Rym_08	22.10	5.08	12.10	2.70	0.90	2.30	1.60	1.50	3.39	1.69	0.3913	1.4375	1.5333	2.0059	4.4815

Rym_09	22.50	5.37	12.90	2.70	0.90	2.20	1.50	1.40	3.82	1.55	0.4091	1.4667	1.5714	2.4645	4.7778
Rym_10	22.60	5.15	12.30	2.70	0.90	2.50	1.60	1.50	3.60	1.55	0.3600	1.5625	1.6667	2.3226	4.5556
Rym_11	23.00	5.29	11.80	2.50	1.00	2.30	1.60	1.50	3.60	1.69	0.4348	1.4375	1.5333	2.1302	4.7200
Rym_12	22.40	5.37	12.30	2.60	1.00	2.20	1.50	1.50	3.82	1.55	0.4545	1.4667	1.4667	2.4645	4.7308
Rym_13	22.00	5.01	12.20	2.40	0.80	2.40	1.50	1.40	3.60	1.41	0.3333	1.6000	1.7143	2.5532	5.0833
Rym_14	22.10	5.26	12.90	2.70	0.90	2.50	1.60	1.50	3.71	1.55	0.3600	1.5625	1.6667	2.3935	4.7778
Rym_15	22.20	5.26	12.00	2.60	0.90	2.40	1.50	1.40	3.71	1.55	0.3750	1.6000	1.7143	2.3935	4.6154
Rym_16	20.16	4.80	12.01	2.73	1.04	2.20	1.49	1.45	3.37	1.43	0.4727	1.4765	1.5172	2.3566	4.3993
Rym_17	18.74	4.87	11.84	2.60	0.9	2.28	1.54	1.50	3.30	1.57	0.3947	1.4805	1.5200	2.1019	4.5538
Rym_18	19.04	4.98	12.39	2.52	1.09	2.49	1.45	1.41	3.63	1.35	0.4378	1.7172	1.7660	2.6889	4.9167
Rjhm	20.01	5.44	12.65	2.66	1.02	2.34	1.47	1.44	3.75	1.69	0.4359	1.5918	1.6250	2.2189	4.7556
Rjpm	20.48	5.42	12.20	2.75	0.98	2.48	1.62	1.35	3.75	1.67	0.3952	1.5309	1.8370	2.2455	4.4364

Note: The unit for the measurements is in millimeters. The sample codes are interpreted as below: Rahm–holotype male of *Ranacris albicornis*, Rapm–paratype male of *R albicornis*, Ram–male of *Ranacris albicornis*, Ram_mean–mean of male *R. albicornis*, Ryhm–holotype male of *R. yunnanensis*, Rypm–paratype male of *R. yunnanensis*, Rym–male of *R. yunnanensis*, Rym_mean–mean of male *R. yunnanensis*, Rjhm–holotype male of *R. jinpingensis*, Rjpm–paratype male of *R. jinpingensis*, Rjm_mean–mean of male *R. jinpingensis*, Ram-Rym, Ram-Rjm and Rym-Rjm–species pairs for performing two-sample Wilcoxon test ("Mann-Whitney " test). The acronyms for measurements and ratio indices are as follows: BL–Body length, PNL–Pronotum length, HFL–Hind femur length, HFW–Hind femur width, IOD–Interocular distance, LDE–Longitudinal diameter of eyes, TDE–Transverse diameter of eyes, SOFL–Length of subocular furrow, PZL–Prozona length, MZL–Metazona length, IOD/LDE–Ratio of IOD to LDE, LDE/TDE–Ratio of LDE to TDE, LDE/SOFL–Ratio of LDE to SOFL, PZL/MZL–Ratio of PZL to MZL, HFL/HFW–Ratio of HFL to HFW.

Table S2. Measurements of female *Ranacris* spp.

Sample code	BL	PNL	HFL	HFW	IOD	LDE	TDE	SOFL	PZL	MZL	IOD/ LDE	LDE/ TDE	LDE/ SOFL	PZL/ MZL	HFL/ HFW
Raf_01	26.96	7.52	17.69	3.63	1.53	2.76	1.68	2.32	5.18	2.34	0.5543	1.6429	1.1897	2.2137	4.8733
Raf_02	29.21	7.78	18.26	3.96	1.50	2.96	1.97	2.53	5.18	2.60	0.5068	1.5025	1.1700	1.9923	4.6111
Raf_03	27.36	7.32	17.38	3.84	1.40	2.97	1.69	2.33	4.81	2.51	0.4714	1.7574	1.2747	1.9163	4.5260
Raf_04	24.65	6.96	15.39	3.58	1.35	2.66	1.54	2.57	4.63	2.33	0.5075	1.7273	1.0350	1.9871	4.2989
Raf_05	23.18	6.63	15.57	3.68	1.40	2.72	1.54	2.43	4.44	2.19	0.5147	1.7662	1.1193	2.0274	4.2310
Rypf_01	26.50	6.36	14.90	3.20	1.35	2.50	1.60	1.80	4.40	1.96	0.5400	1.5625	1.3889	2.2449	4.6562
Rypf_02	28.00	7.12	15.60	3.60	1.50	2.70	1.60	2.10	4.60	2.52	0.5556	1.6875	1.2857	1.8254	4.3333
Rypf_03	28.80	7.14	15.80	3.60	1.30	2.70	1.60	2.10	4.90	2.24	0.4815	1.6875	1.2857	2.1875	4.3889

Ryf_01	26.00	6.98	15.10	3.20	1.40	2.60	1.70	1.90	4.60	2.38	0.5385	1.5294	1.3684	1.9328	4.7188
Ryf_02	29.00	7.18	16.10	3.50	1.50	2.70	1.60	2.00	4.80	2.38	0.5556	1.6875	1.3500	2.0168	4.6000
Ryf_03	28.00	6.74	15.10	3.30	1.40	2.60	1.60	2.00	4.50	2.24	0.5385	1.6250	1.3000	2.0089	4.5758
Ryf_04	27.60	6.68	15.30	3.40	1.30	2.70	1.60	1.80	4.30	2.38	0.4815	1.6875	1.5000	1.8067	4.5000
Ryf_05	27.90	6.54	14.80	3.30	1.30	2.50	1.60	1.90	4.30	2.24	0.5200	1.5625	1.3158	1.9196	4.4848
Ryf_06	27.60	6.74	14.90	3.30	1.40	2.60	1.70	1.90	4.50	2.24	0.5385	1.5294	1.3684	2.0089	4.5152
Ryf_07	28.20	6.88	15.70	3.40	1.30	2.70	1.70	1.90	4.50	2.38	0.4815	1.5882	1.4211	1.8908	4.6176
Ryf_08	27.50	6.98	14.80	3.50	1.20	2.60	1.50	1.90	4.60	2.38	0.4615	1.7333	1.3684	1.9328	4.2286
Ryf_09	28.20	6.74	14.80	3.30	1.20	2.60	1.60	2.00	4.50	2.24	0.4615	1.6250	1.3000	2.0089	4.4848
Ryf_10	27.80	6.84	15.30	3.40	1.30	2.60	1.70	1.90	4.60	2.24	0.5000	1.5294	1.3684	2.0536	4.5000
Ryf_11	27.50	6.30	14.20	3.10	1.10	2.50	1.60	1.80	4.20	2.10	0.4400	1.5625	1.3889	2.0000	4.5806
Ryf_12	26.00	5.92	14.00	3.00	1.20	2.50	1.50	1.80	4.10	1.82	0.4800	1.6667	1.3889	2.2527	4.6667
Ryf_13	28.60	6.70	14.90	3.50	1.30	2.60	1.60	1.80	4.60	2.10	0.5000	1.6250	1.4444	2.1905	4.2571
Ryf_14	25.81	6.08	14.67	3.32	1.32	2.49	1.42	2.09	4.00	2.08	0.5301	1.7535	1.1914	1.9231	4.4187
Ryf_15	24.87	6.44	15.28	3.50	1.23	2.54	1.49	2.21	4.50	1.94	0.4843	1.7047	1.1493	2.3196	4.3657
Rjpf_01	24.18	6.90	14.98	3.48	1.45	2.48	1.51	1.97	4.48	2.42	0.5847	1.6424	1.2589	1.8512	4.3046
Rjpf_02	23.76	6.71	14.48	3.13	1.37	2.57	1.62	1.73	4.50	2.21	0.5331	1.5864	1.4855	2.0362	4.6262

Note: The unit for the measurements is in millimeters. The sample codes are interpreted as below. Raf–female of *R. albicornis*, Raf_mean–mean of female *R. albicornis*, Rypf–paratype female of *R. yunnanensis*, Ryf–female of *R. yunnanensis*, Rjpf–paratype female of *R. jinpingensis,* Rjf_mean–mean of female *R. jinpingensis*. Ram-Rym, Ram-Rjm and Rym-Rjm–species pairs for performing two-sample Wilcoxon test ("Mann-Whitney " test). The acronyms for measurements ratio indices are as follows: BL–Body length, PNL–Pronotum length, HFL–Hind femur length, HFW–Hind femur width, IOD–Interocular distance, LDE–Longitudinal diameter of eyes, TDE–Transverse diameter of eyes, SOFL–Length of subocular furrow, PZL–Prozona length, MZL–Metazona length, IOD/LDE–Ratio of IOD to LDE, LDE/TDE–Ratio of LDE to TDE, LDE/SOFL–Ratio of LDE to SOFL, PZL/MZL–Ratio of PZL to MZL, HFL/HFW–Ratio of HFL to HFW.

Table S3. Statistics of the measurements and indices of male *Ranacris* spp.

Species	Number of measured samples	BL mean±sd	BL extrema	PNL mean±sd	PNL extrema	HFL mean±sd	HFL extrema	HFW mean±sd	HFW extrema	IOD mean±sd	IOD extrema
R. albicornis	15	20.15±3.63	17.41–23.03	5.15±0.52	4.73–5.70	13.08±1.13	12.25–13.98	2.84±0.37	2.55–3.30	1.05±0.18	0.90–1.20
R. yunnanensis	24	22.20±2.41	18.74–24.00	5.11±0.36	4.80–5.40	12.25±0.58	11.80–12.90	2.61±0.25	2.20–2.80	0.94±0.14	0.80–1.10
R. jinpingensis	2	20.25±0.65	20.01–20.48	5.43±0.03	5.42–5.44	12.43±0.62	12.20–12.65	2.71±0.12	2.66–2.75	1.00±0.06	0.98–1.02

Species	Number of measured samples	LDE mean±sd	LDE extrema	TDE mean±sd	TDE extrema	SOFL mean±sd	SOFL extrema	PZL mean±sd	PZL extrema	MZL mean±sd	MZL extrema
R. albicornis	15	2.60±0.16	2.43–2.70	1.67±0.21	1.49–1.90	1.65±0.16	1.50–1.85	3.67±0.39	3.32–4.00	1.48±0.24	1.20–1.70
R. yunnanensis	24	2.37±0.31	2.10–2.90	1.53±0.10	1.45–1.60	1.46±0.10	1.40–1.50	3.59±0.30	3.29–3.82	1.51±0.25	1.13–1.69
R. jinpingensis	2	2.41±0.19	2.34–2.48	1.54±0.21	1.47–1.62	1.40±0.12	1.35–1.44	3.75±0.00	3.75-3.75	1.68±0.03	1.67–1.69

Species	Number of measured samples	IOD/LDE mean±sd	IOD/LDE extrema	LDE/TDE mean±sd	LDE/TDE extrema	LDE/SOFL mean±sd	LDE/SOFL extrema	PZL/MZL mean±sd	PZL/MZL extrema	HFL/HFW mean±sd	HFL/HFW extrema
R. albicornis	15	0.41±0.07	0.33–0.46	1.56±0.18	1.32–1.69	1.57±0.15	1.41–1.69	2.49±0.44	2.18–3.08	4.61±0.54	4.00–5.03
R. yunnanensis	24	0.40±0.08	0.31–0.47	1.55±0.18	1.40–1.81	1.63±0.23	1.40–1.93	2.39±0.53	2.01–3.28	4.70±0.49	4.39–5.55
R. jinpingensis	2	0.42±0.06	0.40–0.44	1.56±0.08	1.53–1.59	1.73±0.29	1.63–1.82	2.23±0.04	2.22–2.25	4.60±0.44	4.42–4.76

Note. The unit for the measurements is in millimeters. The acronyms for measurements and indices are as follows: BL–Body length, PNL–Pronotum length, HFL–Hind femur length, HFW–Hind femur width, IOD–Interocular distance, LDE–Longitudinal diameter of eyes, TDE–Transverse diameter of eyes, SOFL–Length of subocular furrow, PZL–Prozona length, MZL–Metazona length, IOD/LDE–Ratio of IOD to LDE, LDE/TDE–Ratio of LDE to TDE, LDE/SOFL–Ratio of LDE to SOFL, PZL/MZL–Ratio of PZL to MZL, HFL/HFW–Ratio of HFL to HFW.

Table S4. Statistics of the measurements and indices of female *Ranacris* spp.

Species	Number of measured samples	BL mean±sd	BL extrema	PNL mean±sd	PNL extrema	HFL mean±sd	HFL extrema	HFW mean±sd	HFW extrema	IOD mean±sd	IOD extrema
R. albicornis	5	26.27±4.65	23.18-29.21	7.24±0.89	6.63-7.78	16.86±2.55	15.39-18.26	3.74±0.31	3.58-3.96	1.44±0.15	1.35-1.53
R. yunnanensis	18	27.44±2.23	24.87-29.00	6.69±0.70	5.92-7.18	15.07±1.04	14.00-16.10	3.36±0.32	3.00-3.60	1.31±0.20	1.10-1.50
R. jinpingensis	2	23.97±0.58	23.76-24.18	6.81±0.26	6.71-6.90	14.73±0.69	14.48-14.98	3.31±0.49	3.13-3.48	1.41±0.11	1.37-1.45

Species	Number of measured samples	LDE mean±sd	LDE extrema	TDE mean±sd	TDE extrema	SOFL mean±sd	SOFL extrema	PZL mean±sd	PZL extrema	MZL mean±sd	MZL extrema
R. albicornis	5	2.81±0.28	2.66-2.97	1.68±0.34	1.54-1.97	2.44±0.22	2.32-2.57	4.85±0.65	4.44-5.18	2.39±0.32	2.19-2.60
R. yunnanensis	18	2.60±0.15	2.49-2.70	1.60±0.15	1.42-1.70	1.94±0.24	1.80-2.21	4.47±0.44	4.00-4.90	2.21±0.36	1.82-2.52
R. jinpingensis	2	2.53±0.12	2.48-2.57	1.57±0.15	1.51-1.62	1.85±0.33	1.73-1.97	4.49±0.03	4.48-4.50	2.32±0.29	2.21-2.42

Species	Number of measured samples	IOD/LDE mean±sd	IOD/LDE extrema	LDE/TDE mean±sd	LDE/TDE extrema	LDE/SOFL mean±sd	LDE/SOFL extrema	PZL/MZL mean±sd	PZL/MZL extrema	HFL/HFW mean±sd	HFL/HFW extrema
R. albicornis	5	0.51±0.06	0.47-0.55	1.68±0.22	1.50-1.77	1.16±0.17	1.04-1.27	2.03±0.22	1.92-2.21	4.51±0.50	4.23-4.87
R. yunnanensis	18	0.50±0.07	0.44-0.56	1.63±0.14	1.53-1.75	1.34±0.17	1.15-1.50	2.03±0.30	1.81-2.32	4.49±0.27	4.23-4.72
R. jinpingensis	2	0.56±0.07	0.53-0.58	1.61±0.08	1.59-1.64	1.37±0.31	1.26-1.49	1.94±0.26	1.85-2.04	4.47±0.45	4.30-4.63

Note. The unit and acronyms for the measurements and indices are the same as those in Table S1.

Table S5. Results of discriminant analysis of male *Ranacris* spp. when the sample of *R. jinpingensis* was assigned to *R. albicornis*.

Sample	species	posterior probability of discrimination (based on measurements)		posterior probability of discrimination (based on ratio indices)		posterior probability of discrimination (based on full data)	
		Ram	Rym	Ram	Rym	Ram	Rym
Rahm	Ram	9.999962e-01	3.845133e-06	0.2155448	0.7844552	9.999899e-01	1.006968e-05
Rapm_01	Ram	9.999922e-01	7.841263e-06	0.8847386	0.1152614	9.999999e-01	6.736127e-08
Rapm_02	Ram	9.999107e-01	8.926544e-05	0.4095451	0.5904549	9.998335e-01	1.665438e-04
Ram_01	Ram	9.999998e-01	1.938844e-07	0.4323730	0.5676270	1.000000e+00	9.097663e-10
Ram_02	Ram	9.978819e-01	2.118107e-03	0.4845124	0.5154876	9.994442e-01	5.557858e-04
Ram_03	Ram	9.999998e-01	1.773510e-07	0.4040045	0.5959955	1.000000e+00	2.307656e-08
Ram_04	Ram	9.998252e-01	1.748271e-04	0.5339454	0.4660546	9.999646e-01	3.536394e-05
Ram_05	Ram	9.992849e-01	7.151239e-04	0.4332419	0.5667581	9.970780e-01	2.922012e-03
Ram_06	Ram	9.999998e-01	1.905822e-07	0.4997489	0.5002511	1.000000e+00	3.931190e-08
Ram_07	Ram	9.993561e-01	6.439371e-04	0.6648828	0.3351172	9.991968e-01	8.032214e-04
Ram_08	Ram	9.999967e-01	3.343950e-06	0.4797648	0.5202352	9.999982e-01	1.806476e-06
Ram_09	Ram	9.999783e-01	2.171683e-05	0.3977605	0.6022395	9.999938e-01	6.208397e-06
Ram_10	Ram	9.998333e-01	1.666911e-04	0.5962312	0.4037688	9.999366e-01	6.336419e-05
Ram_11	Ram	1.000000e+00	4.127863e-09	0.3880118	0.6119882	1.000000e+00	1.057026e-09
Ram_12	Ram	9.999869e-01	1.308077e-05	0.7091456	0.2908544	9.999984e-01	1.648802e-06
Ryhm	Rym	2.605916e-06	9.999974e-01	0.4910428	0.5089572	4.329552e-07	9.999996e-01
Rypm_01	Rym	7.791452e-07	9.999992e-01	0.1123541	0.8876459	6.347285e-07	9.999994e-01
Rypm_02	Rym	5.695449e-05	9.999430e-01	0.2374503	0.7625497	7.382638e-06	9.999926e-01
Rypm_03	Rym	3.496052e-05	9.999650e-01	0.4357312	0.5642688	2.320958e-06	9.999977e-01
Rypm_04	Rym	2.345172e-06	9.999977e-01	0.3503569	0.6496431	1.183603e-06	9.999988e-01
Rypm_05	Rym	7.398657e-04	9.992601e-01	0.2767874	0.7232126	8.478415e-06	9.999915e-01
Rym_01	Rym	7.561134e-07	9.999992e-01	0.4688920	0.5311080	3.983995e-08	1.000000e+00
Rym_02	Rym	5.049501e-07	9.999995e-01	0.2462863	0.7537137	2.246811e-07	9.999998e-01
Rym_03	Rym	4.602904e-07	9.999995e-01	0.5513280	0.4486720	6.828816e-07	9.999993e-01
Rym_04	Rym	1.927936e-06	9.999981e-01	0.6797124	0.3202876	1.245573e-07	9.999999e-01
Rym_05	Rym	8.013722e-06	9.999920e-01	0.3304589	0.6695411	5.221048e-06	9.999948e-01
Rym_06	Rym	1.090676e-06	9.999989e-01	0.3268313	0.6731687	7.577104e-07	9.999992e-01
Rym_07	Rym	3.910868e-08	1.000000e+00	0.2673310	0.7326690	1.664185e-08	1.000000e+00
Rym_08	Rym	7.104732e-05	9.999290e-01	0.3479639	0.6520361	7.843616e-05	9.999216e-01
Rym_09	Rym	1.481728e-04	9.998518e-01	0.3478214	0.6521786	2.520188e-05	9.999748e-01
Rym_10	Rym	1.471741e-04	9.998528e-01	0.3393610	0.6606390	3.239788e-05	9.999676e-01
Rym_11	Rym	3.791807e-06	9.999962e-01	0.3149325	0.6850675	5.381367e-07	9.999995e-01
Rym_12	Rym	4.992848e-05	9.999501e-01	0.5246830	0.4753170	1.096102e-06	9.999989e-01
Rym_13	Rym	3.226239e-08	1.000000e+00	0.2109460	0.7890540	1.113863e-08	1.000000e+00
Rym_14	Rym	5.061044e-02	9.493896e-01	0.2876323	0.7123677	1.935817e-02	9.806418e-01
Rym_15	Rym	1.247341e-06	9.999988e-01	0.3168658	0.6831342	3.594074e-07	9.999996e-01
Rym_16	Rym	1.087119e-03	9.989129e-01	0.5577714	0.4422286	8.552921e-05	9.999145e-01
Rym_17	Rym	9.079487e-03	9.909205e-01	0.4100845	0.5899155	7.492481e-03	9.925075e-01
Rym_18	Rym	4.438879e-01	5.561121e-01	0.3558293	0.6441707	3.483816e-01	6.516184e-01

Rjhm	Ram	7.857030e-01	2.142970e-01	0.3548355	0.6451645	5.581680e-01	4.418320e-01
Rjpm	Ram	4.752684e-01	5.247316e-01	0.1619448	0.8380552	9.976903e-01	2.309671e-03

Table S6. Results of discriminant analysis of female *Ranacris* spp. when the sample of *R. jinpingensis* was assigned to *R. albicornis*.

Sample	species	posterior probability of discrimination (based on measurements)		posterior probability of discrimination (based on ratio indices)		posterior probability of discrimination (based on full data)	
		Raf	Ryf	Raf	Ryf	Raf	Ryf
Raf_01	Raf	9.998485e-01	1.515204e-04	0.811416875	0.1885831	9.999998e-01	2.191990e-07
Raf_02	Raf	9.998604e-01	1.396381e-04	0.630411108	0.3695889	9.999282e-01	7.175416e-05
Raf_03	Raf	9.997121e-01	2.879147e-04	0.367320989	0.6326790	9.999991e-01	8.632617e-07
Raf_04	Raf	9.996009e-01	3.990752e-04	0.870289279	0.1297107	1.000000e+00	1.271322e-08
Raf_05	Raf	9.999981e-01	1.920222e-06	0.641297077	0.3587029	9.999999e-01	7.209212e-08
Rypf_01	Ryf	2.846543e-05	9.999715e-01	0.072808509	0.9271915	5.909347e-07	9.999994e-01
Rypf_02	Ryf	1.054727e-03	9.989453e-01	0.410993376	0.5890066	3.374914e-08	1.000000e+00
Rypf_03	Ryf	4.244000e-05	9.999576e-01	0.096688651	0.9033113	2.750404e-07	9.999997e-01
Ryf_01	Ryf	6.553978e-01	3.446022e-01	0.252776211	0.7472238	3.740374e-03	9.962596e-01
Ryf_02	Ryf	2.351370e-05	9.999765e-01	0.291947505	0.7080525	6.434622e-08	9.999999e-01
Ryf_03	Ryf	3.001445e-06	9.999970e-01	0.339845818	0.6601542	5.510320e-08	9.999999e-01
Ryf_04	Ryf	1.613271e-04	9.998387e-01	0.032503422	0.9674966	1.860512e-04	9.998139e-01
Ryf_05	Ryf	2.937111e-07	9.999997e-01	0.212172126	0.7878279	9.254313e-09	1.000000e+00
Ryf_06	Ryf	4.356300e-05	9.999564e-01	0.108689073	0.8913109	9.034576e-06	9.999910e-01
Ryf_07	Ryf	5.337372e-04	9.994663e-01	0.076581269	0.9234187	1.617402e-05	9.999838e-01
Ryf_08	Ryf	7.703687e-05	9.999230e-01	0.040893320	0.9591067	1.807418e-08	1.000000e+00
Ryf_09	Ryf	1.293945e-06	9.999987e-01	0.132853126	0.8671469	1.739767e-08	1.000000e+00
Ryf_10	Ryf	1.971847e-04	9.998028e-01	0.060055256	0.9399447	8.029506e-06	9.999920e-01
Ryf_11	Ryf	9.163682e-08	9.999999e-01	0.042316697	0.9576833	4.461642e-11	1.000000e+00
Ryf_12	Ryf	3.201058e-07	9.999997e-01	0.052639676	0.9473603	9.594699e-13	1.000000e+00
Ryf_13	Ryf	4.975763e-08	1.000000e+00	0.007751268	0.9922487	4.786110e-09	1.000000e+00
Ryf_14	Ryf	1.465745e-05	9.999853e-01	0.691107886	0.3088921	4.404513e-07	9.999996e-01
Ryf_15	Ryf	4.043464e-01	5.956536e-01	0.298038760	0.7019612	6.846861e-03	9.931531e-01
Rjpf_01	Raf	9.937628e-01	6.237236e-03	0.498815079	0.5011849	9.992066e-01	7.933978e-04
Rjpf_02	Raf	9.971987e-01	2.801254e-03	0.037592894	0.9624071	9.999955e-01	4.455051e-06

Table S7. Results of discriminant analysis of male *Ranacris* spp. when the sample of *R. jinpingensis* was assigned to *R. yunnanensis*.

Sample	species	posterior probability of discrimination (based on measurements)		posterior probability of discrimination (based on ratio indices)		posterior probability of discrimination (based on full data)	
		Ram	Rym	Ram	Rym	Ram	Rym
Rahm	Ram	1.000000e+00	3.009025e-09	0.09196830	0.90803170	1.000000e+00	6.429581e-13
Rapm_01	Ram	1.000000e+00	7.524686e-12	0.93806459	0.06193541	1.000000e+00	2.640658e-17
Rapm_02	Ram	1.000000e+00	4.314710e-08	0.36457544	0.63542456	1.000000e+00	2.055866e-11
Ram_01	Ram	1.000000e+00	3.837915e-15	0.63486483	0.36513517	1.000000e+00	1.899245e-17
Ram_02	Ram	1.000000e+00	8.604458e-11	0.54321533	0.45678467	1.000000e+00	5.128502e-14
Ram_03	Ram	1.000000e+00	1.539100e-12	0.28394882	0.71605118	1.000000e+00	5.560966e-18
Ram_04	Ram	1.000000e+00	6.061612e-10	0.70627262	0.29372738	1.000000e+00	6.855677e-13
Ram_05	Ram	9.999991e-01	8.597653e-07	0.45406234	0.54593766	1.000000e+00	2.018086e-09
Ram_06	Ram	1.000000e+00	1.925807e-08	0.40251949	0.59748051	1.000000e+00	7.365142e-12
Ram_07	Ram	9.999999e-01	8.650907e-08	0.70608565	0.29391435	1.000000e+00	7.200087e-10
Ram_08	Ram	1.000000e+00	6.573279e-09	0.42607717	0.57392283	1.000000e+00	1.232105e-13
Ram_09	Ram	1.000000e+00	3.920213e-09	0.31519311	0.68480689	1.000000e+00	9.136551e-11
Ram_10	Ram	9.999996e-01	3.772716e-07	0.62219961	0.37780039	1.000000e+00	9.750347e-10
Ram_11	Ram	1.000000e+00	2.370433e-12	0.28593930	0.71406070	1.000000e+00	6.716381e-16
Ram_12	Ram	1.000000e+00	1.034497e-10	0.72060130	0.27939870	1.000000e+00	6.426984e-15
Ryhm	Rym	1.024004e-10	1.000000e+00	0.57221861	0.42778139	3.071170e-13	1.000000e+00
Rypm_01	Rym	1.083536e-13	1.000000e+00	0.05278147	0.94721853	7.004481e-16	1.000000e+00
Rypm_02	Rym	9.070218e-09	1.000000e+00	0.17410808	0.82589192	2.035576e-09	1.000000e+00
Rypm_03	Rym	6.834497e-12	1.000000e+00	0.34598192	0.65401808	6.488497e-17	1.000000e+00
Rypm_04	Rym	2.508744e-11	1.000000e+00	0.15975932	0.84024068	6.226135e-14	1.000000e+00
Rypm_05	Rym	1.409016e-07	9.999999e-01	0.13070419	0.86929581	4.448424e-13	1.000000e+00
Rym_01	Rym	3.545710e-12	1.000000e+00	0.47641650	0.52358350	2.387525e-13	1.000000e+00
Rym_02	Rym	1.288353e-11	1.000000e+00	0.10017423	0.89982577	1.310388e-16	1.000000e+00
Rym_03	Rym	6.140530e-08	9.999999e-01	0.68935661	0.31064339	3.280385e-17	1.000000e+00
Rym_04	Rym	1.700868e-10	1.000000e+00	0.74909121	0.25090879	3.186547e-13	1.000000e+00
Rym_05	Rym	9.103886e-14	1.000000e+00	0.19833914	0.80166086	1.777888e-16	1.000000e+00
Rym_06	Rym	4.491948e-14	1.000000e+00	0.18544923	0.81455077	7.719208e-17	1.000000e+00
Rym_07	Rym	6.101846e-14	1.000000e+00	0.14907183	0.85092817	1.730823e-17	1.000000e+00
Rym_08	Rym	1.358398e-08	1.000000e+00	0.23446166	0.76553834	8.115311e-13	1.000000e+00
Rym_09	Rym	6.525440e-13	1.000000e+00	0.27762270	0.72237730	9.168523e-17	1.000000e+00
Rym_10	Rym	2.485103e-08	1.000000e+00	0.24937825	0.75062175	1.267783e-08	1.000000e+00
Rym_11	Rym	1.551891e-11	1.000000e+00	0.18468889	0.81531111	2.205076e-16	1.000000e+00
Rym_12	Rym	2.226516e-11	1.000000e+00	0.51982303	0.48017697	2.512155e-15	1.000000e+00
Rym_13	Rym	1.935864e-14	1.000000e+00	0.17552405	0.82447595	2.355151e-16	1.000000e+00
Rym_14	Rym	5.473974e-05	9.999453e-01	0.21701350	0.78298650	1.237166e-05	9.999876e-01
Rym_15	Rym	8.846195e-16	1.000000e+00	0.19944704	0.80055296	1.445670e-18	1.000000e+00
Rym_16	Rym	4.564703e-07	9.999995e-01	0.43870694	0.56129306	3.748770e-11	1.000000e+00
Rym_17	Rym	5.283072e-05	9.999472e-01	0.34249988	0.65750012	4.666020e-07	9.999995e-01
Rym_18	Rym	1.034140e-04	9.998966e-01	0.21739519	0.78260481	9.663701e-08	9.999999e-01

Rjhm	Rym	2.100447e-07	9.999998e-01	0.21128120	0.78871880	1.697902e-13	1.000000e+00
Rjpm	Rym	2.314674e-10	1.000000e+00	0.03209080	0.96790920	1.189529e-15	1.000000e+00

Table S8. Results of discriminant analysis of female *Ranacris* spp. when the sample of *R. jinpingensis* was assigned to *R. yunnanensis*.

Sample	species	posterior probability of discrimination (based on measurements)		posterior probability of discrimination (based on ratio indices)		posterior probability of discrimination (based on full data)	
		Raf	Ryf	Raf	Ryf	Raf	Ryf
Raf_01	Raf	9.999901e-01	9.919262e-06	9.471704e-01	0.05282964	1.000000e+00	1.533115e-12
Raf_02	Raf	1.000000e+00	2.056671e-11	8.635997e-01	0.13640026	1.000000e+00	2.362329e-15
Raf_03	Raf	1.000000e+00	7.267537e-09	5.927229e-01	0.40727714	1.000000e+00	2.101337e-13
Raf_04	Raf	9.999995e-01	5.155318e-07	9.888966e-01	0.01110337	1.000000e+00	9.668999e-17
Raf_05	Raf	9.999999e-01	7.192706e-08	6.788830e-01	0.32111703	1.000000e+00	2.097150e-15
Rypf_01	Ryf	1.200177e-11	1.000000e+00	1.162116e-03	0.99883788	7.286020e-18	1.000000e+00
Rypf_02	Ryf	2.288586e-07	9.999998e-01	1.476192e-02	0.98523808	2.028235e-15	1.000000e+00
Rypf_03	Ryf	3.860823e-06	9.999961e-01	2.354446e-02	0.97645554	2.767461e-10	1.000000e+00
Ryf_01	Ryf	2.895110e-07	9.999997e-01	1.970947e-02	0.98029053	4.270308e-15	1.000000e+00
Ryf_02	Ryf	1.625938e-08	1.000000e+00	1.273698e-02	0.98726302	3.664921e-13	1.000000e+00
Ryf_03	Ryf	3.774506e-09	1.000000e+00	5.066303e-02	0.94933697	1.463075e-15	1.000000e+00
Ryf_04	Ryf	1.008745e-09	1.000000e+00	4.371711e-04	0.99956283	6.895402e-13	1.000000e+00
Ryf_05	Ryf	3.790041e-13	1.000000e+00	1.718870e-02	0.98281130	3.225627e-18	1.000000e+00
Ryf_06	Ryf	3.626279e-10	1.000000e+00	1.320931e-03	0.99867907	2.080811e-18	1.000000e+00
Ryf_07	Ryf	2.255912e-07	9.999998e-01	7.642584e-03	0.99235742	5.755566e-11	1.000000e+00
Ryf_08	Ryf	4.932006e-12	1.000000e+00	1.978051e-03	0.99802195	2.902597e-23	1.000000e+00
Ryf_09	Ryf	8.128703e-09	1.000000e+00	1.039583e-01	0.89604174	7.817487e-17	1.000000e+00
Ryf_10	Ryf	1.071350e-09	1.000000e+00	2.173187e-03	0.99782681	3.482093e-15	1.000000e+00
Ryf_11	Ryf	1.284996e-12	1.000000e+00	1.843820e-02	0.98156180	3.170935e-17	1.000000e+00
Ryf_12	Ryf	1.098956e-10	1.000000e+00	1.078103e-02	0.98921897	9.534704e-23	1.000000e+00
Ryf_13	Ryf	2.359760e-14	1.000000e+00	9.966760e-06	0.99999003	1.242187e-19	1.000000e+00
Ryf_14	Ryf	3.645046e-08	1.000000e+00	6.305467e-01	0.36945329	6.471222e-15	1.000000e+00
Ryf_15	Ryf	3.239562e-02	9.676044e-01	5.014047e-01	0.49859530	1.919901e-08	1.000000e+00
Rjpf_01	Raf	4.589041e-10	1.000000e+00	8.480544e-03	0.99151946	1.099065e-18	1.000000e+00
Rjpf_02	Raf	1.599790e-08	1.000000e+00	1.299037e-04	0.99987010	4.641389e-13	1.000000e+00
